# Supplementary material for: Estimated projection of oral squamous cell carcinoma annual incidence from twenty years registry data: a retrospective cross-sectional study in Indonesia
Source: PeerJ. 2023 Aug 29;11:e15911. doi: 10.7717/peerj.15911 (PMC10473041; doi:10.7717/peerj.15911)
Supplement: Supplemental Information 2 [file peerj-11-15911-s002.pdf]

This README_Dataset_OSCC_Indonesian_Projection.txt file was generated on December 11, 2022, by Nur Rahadiani

GENERAL INFORMATION

1. Title of Dataset: "Data from: Estimated Projection of Oral Squamous Cell Carcinoma Annual Incidence Applied to Twenty Years Registry Data in the Main Referral Hospital in Indonesia."

2. Author Information
Corresponding Investigator
Name: Nur Rahadiani, MD., Pathologist, Ph.D.
Institution: Department of Anatomical Pathology, Faculty of Medicine Universitas Indonesia, Dr. Cipto Mangunkusumo Hospital, Central Jakarta, DKI Jakarta, Indonesia
Email: nur.rahadiani@ui.ac.id
ORCID iD: 0000-0003-2342-8133

Co-investigator 1
Name: Muhammad Habiburrahman, MD.
Institution: Faculty of Medicine Universitas Indonesia, Dr. Cipto Mangunkusumo Hospital, Central Jakarta, DKI Jakarta, Indonesia
Email: muhammad.habiburrahman51@ui.ac.id
ORCID iD: 0000-0001-6372-8240

Co-investigator 2
Name: Marini Stephanie, MD., Pathologist
Institution: Department of Anatomical Pathology, Faculty of Medicine Universitas Indonesia, Dr. Cipto Mangunkusumo Hospital, Central Jakarta, DKI Jakarta, Indonesia
Email: marini2sh@gmail.com
ORCID iD: 0000-0002-5743-688X

Co-investigator 3
Name: Diah Rini Handjari, MD., Pathologist, Ph.D.
Institution: Department of Anatomical Pathology, Faculty of Medicine Universitas Indonesia, Dr. Cipto Mangunkusumo Hospital, Central Jakarta, DKI Jakarta, Indonesia
Email: rinidiah@gmail.com
ORCID iD: 0000-0001-9377-0984

Co-investigator 4
Name: Ening Krisnuhoni, MD., Pathologist, MSc.
Institution: Department of Anatomical Pathology, Faculty of Medicine Universitas Indonesia, Dr. Cipto Mangunkusumo Hospital, Central Jakarta, DKI Jakarta, Indonesia
Email: ening.krisnuhoni@yahoo.com
ORCID iD: 0000-0002-3560-5590

3. Date of data collection: from January 2001 to December 2020

4. Geographic location of data collection: DKI Jakarta, Indonesia

5. Funding sources that supported collecting the data: Ministry of Research and Technology/National Agency for Research and Innovation (BRIN) through the Research and Community Service Information System (SIMLITABMAS) and Top Basic Research in University (PDUPT) grant scheme (Grant number: NKB-122, year: 2021).

6. Recommended citation for this dataset: Rahadiani, N et al. (2022), Estimated Projection of Oral Squamous Cell Carcinoma Annual Incidence Applied to Twenty Years Registry Data in the Main Referral Hospital in Indonesia.

DATA & FILE OVERVIEW

1. Description of dataset

These data were generated to measure the incidence rate of OSCC in Indonesia's national tertiary hospital between 2001 and 2020, particularly in young and female patients. The collected data were also analysed to predict the annual trends and projections as well as highlight the demographic, clinicopathological, and histological findings in patients with OSCC based on gender. The dataset has been anonymised by obscuring specific personal identities and detailed socio-demographic information because it contains sensitive and potentially identifying individual information. With this regard, requests to access these data should be made to the Faculty of Medicine, Universitas Indonesia's ethical committee (ec_fkui@yahoo.com).

2. File List:
File Name: Dataset_OSCC_Indonesian_Projection
File Description: 20-year dataset of clinicopathological characteristics of patients with oral squamous cell carcinoma from a single centre in Indonesia between 2001 and 2020

METHODOLOGICAL INFORMATION

This is data from a retrospective cross-sectional study examined OSCC cases treated at Dr. Cipto Mangunkusumo Hospital, Indonesia, and recorded in our institution's twenty-year cancer registry data between January 2001 and December 2020.All patients with OSCC who underwent a histopathological examination became our study sample. Exclusion criteria include missing slides, change of diagnosis following reassessment, and duplicate inputs due to several specimen-taking procedures performed on the same patient. The data from tissue blocks stained with hematoxylin and eosin (H&E), slide archives, and the patient's clinical profile were input to produce valuable clinicopathological information. The authors assessed the pathological diagnosis separately and held discussions to establish the final diagnosis.

DATA-SPECIFIC INFORMATION FOR: Dataset_OSCC_Indonesian_Projection.xlsx

1. Number of variables: 21

2. Number of cases/rows: 1,093

3. List of variables and their definitions:

No_of_patients: Numerical code assigned to respondents; 1 to 1,093
Year_Record: The patient's disease diagnosis year was recorded in the hospital's cancer registry data; 2001 to 2020
Five_Year_Record: The classification of a patient's disease diagnosis year by year over a five-year period; 2001-2005, 2006-2010, 2011-2015, 2016-2020
Age: Patient's age at the time of disease diagnosis; 17 to 99
Age_Group: Grouping of patient's age; 11-20, 21-30, 31-40, 41-50, 51-60, 61-70, 71-80, >80
Young_Old_Patients: Grouping of subject's age using a cut-off 45 y.o. where 'young patient' was categorized as those with age and <45 y.o.; <=45 (Young patient), >45 (Old patient)
Sex: Sex of a patient; Male, Female
Tumor_Sites: The tumour's location where the cancer grew and was diagnosed, as reported using the ICD-10 system; Tongue, Mouth Non-specific, Palate, Gingiva, Lip, Buccal mucosa, FOM
Keratinization: The presence of keratin in tumour pathological specimens as defined using the WHO guideline and reported using ICD-10 code groupings; Yes, No, Non-specific
WHO_Differentiation: The classification of histological differentiation using the WHO guideline; Well, Moderate, Poor, Undifferentiated
Bryne_Score: Degree of cellular differentiation according to the Bryne (1992) grading system; 4 to 16
Bryne_Grading: The categorisation of Bryne score according to the Bryne (1992) grading system; 4-8 (Grade I, Good), 9-12 (Grade II, Moderate), 13-16 (Grade III, Poor)
Specimen_Types: Specimen-taking procedures performed on the patient with OSCC; Biopsy, Excision, Resection
Tumor_Size: The category of the size of the tumour according to the eighth edition AJCC staging manual; T1, T2, T3, T4
Node_Involvement_Status: The presence of lymph node metastasis in patients with the OSCC; N0, N1, N2
Distant_Metastasis_Status: The presence of distant metastasis in patients with the OSCC; M0, M1
TNM_Status: TNM status in the patients who had surgical treatments following the eighth edition AJCC staging manual; Tx Nx Mx
Clinical_Staging: Clinical TNM staging in the patients who had surgical treatments following the eighth edition AJCC staging manual; I, II, III, IV
Degree_Staging: The categorisation of clinical staging according to the degree of disease development; Early Stage (I-II), Advanced Stage (III-IV)
LVI: The presence of lymphovascular invasion in the tumour pathological specimens; Positive, Negative
Margin_Resection: The presence of tumour in the surgical margins for the patient underwent gross tumour resection: Positive, Negative

4. Missing data codes:
NA: (1) missing data because the parameter was measured but was not available in the medical or pathological record; (2) no data because the parameter was not relevant to measure, such as in the biopsy sample; consequently, margin resection cannot be evaluated.

5. Abbreviations used:
y.o.; Years old
ICD-10; International Classification of Diseases 10th Edition

FOM; Floor of mouth
WHO; World Health Organization
OSCC; Oral squamous cell carcinoma
AJCC; American Joint Committee on Cancer
TNM; Tumor, node, metastasis staging system
NA; Not applicable, not available
LVI; Lymphovascular invasion

6. Other relevant information:

To better understand this study and learn more about the interpretation of this dataset, we refer readers to our article at Peer J and view the attached supplementary data.

We also welcome other researchers to contact our corresponding authors if any further questions or inquiries.

-END-
