# Supplementary material for: Estimated projection of oral squamous cell carcinoma annual incidence from twenty years registry data: a retrospective cross-sectional study in Indonesia
Source: PeerJ. 2023 Aug 29;11:e15911. doi: 10.7717/peerj.15911 (PMC10473041; doi:10.7717/peerj.15911)
Supplement: Supplemental Information 4 — Comprehensive analysis of the annual incidence trend of oral squamous cell carcinoma (OSCC) using Joinpoint regression analysis among all patients, stratified by age, sex, and tumor origin sites. [file peerj-11-15911-s004.pdf]

Supplemental Data S2: Trend Analysis using Joinpoint Regression Method

Comprehensive analysis of the annual incidence trend of oral squamous cell carcinoma (OSCC) using Joinpoint regression analysis among all patients, stratified by age, sex, and tumor origin sites.

a. Trend Analysis for Total OSCC Cases

1. Graph

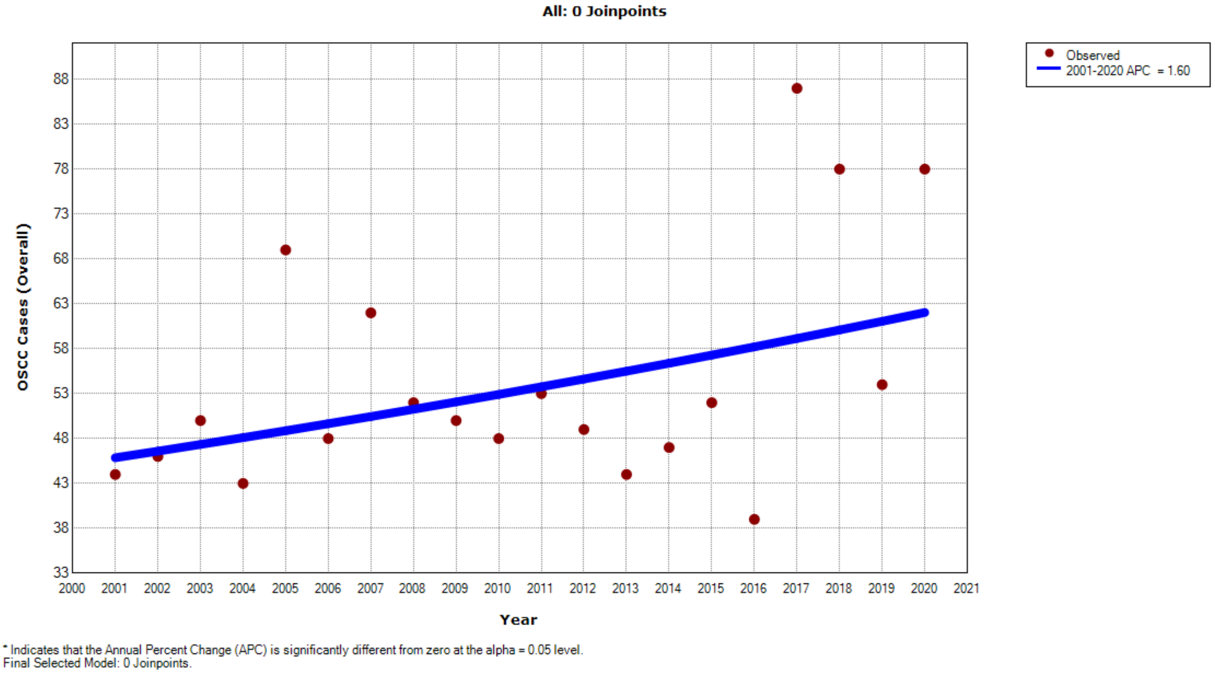

Figure 1. Trend analysis of total OSCC cases from 1,093 Indonesian patients during 2001-2020. Plotted lines represent annual percentage changes (APCs). *Significant deviation in APC from 0 ($p<0.05$) using the permutation model of logarithmically transformed data in Joinpoint regression analysis.

2. The significance test results obtained through the Monte Carlo permutation statistical method were used to identify the best-fitted line segment(s) in the Joinpoint regression analysis, representing significant trend changes (referred to as APC value) in the time series.

Annual Percent Change (APC)

Segment	Lower Endpoint	Upper Endpoint	APC	Lower CI	Upper CI	Test Statistic (t)	Prob > |t|
1	2001.00	2020.00	1.60	-0.07	3.30	2.02	0.059

* Indicates that the Annual Percent Change (APC) is significantly different from zero at the alpha = 0.05 level.

Average Annual Percent Change (AAPC)

Range	Lower Endpoint	Upper Endpoint	AAPC	Lower CI	Upper CI	Test Statistic~	P-Value~
Full Range	2001.00	2020.00	1.60	-0.07	3.30	2.02	0.059

* Indicates that the AAPC is significantly different from zero at the alpha = 0.05 level.
~ If the AAPC is within one segment, the t-distribution is used. Otherwise, the normal (z) distribution is used. See Help to Learn More

b. Trend Analysis for OSCC Cases in Young Patients (≤45 years old)

1. Graph

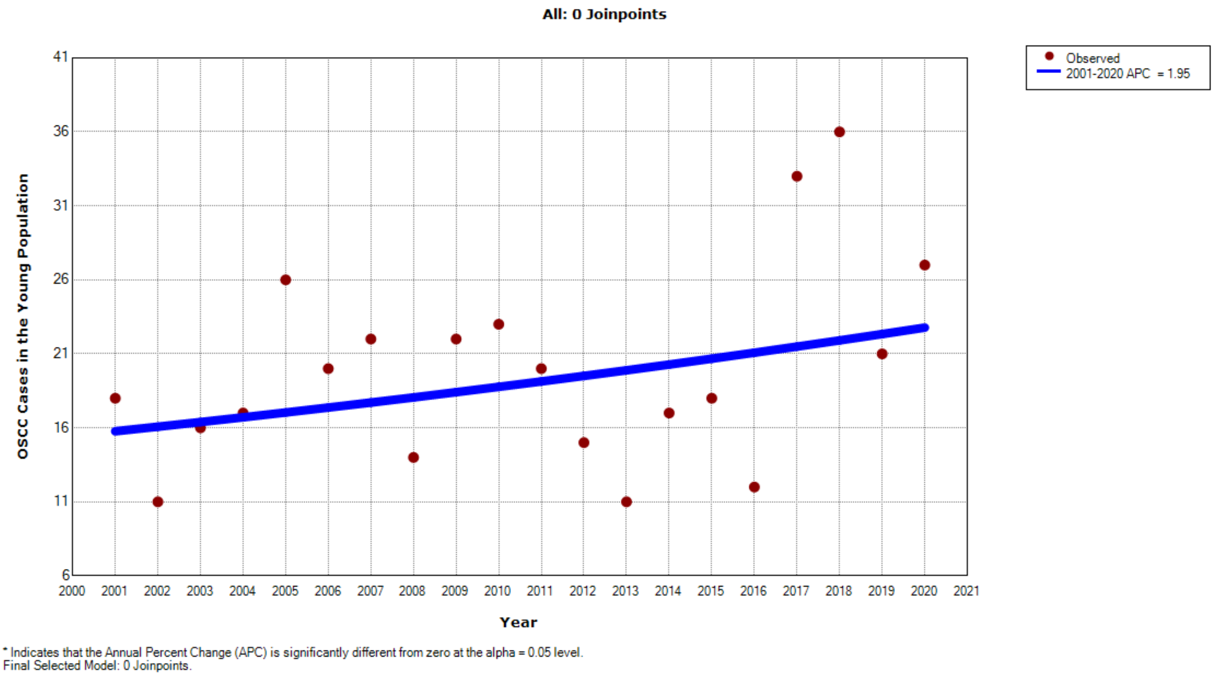

Figure 2. Trend analysis of OSCC cases in young adults (≤45 years old) in Indonesia from 2001 to 2020. The plotted lines represent the annual percentage changes (APCs). *Significant change in APC compared to 0 ($p<0.05$) using the permutation model of logarithmically transformed data in Joinpoint regression analysis.

2. The significance test results obtained through the Monte Carlo permutation statistical method were used to identify the best-fitted line segment(s) in the Joinpoint regression analysis, representing significant trend changes (referred to as APC value) in the time series.

Annual Percent Change (APC)							
Segment	Lower Endpoint	Upper Endpoint	APC	Lower CI	Upper CI	Test Statistic (t)	Prob > |t|
1	2001.00	2020.00	1.95	-0.64	4.61	1.58	0.132
* Indicates that the Annual Percent Change (APC) is significantly different from zero at the alpha = 0.05 level.							

Average Annual Percent Change (AAPC)							
Range	Lower Endpoint	Upper Endpoint	AAPC	Lower CI	Upper CI	Test Statistic~	P-Value~
Full Range	2001.00	2020.00	1.95	-0.64	4.61	1.58	0.132
* Indicates that the AAPC is significantly different from zero at the alpha = 0.05 level. ~ If the AAPC is within one segment, the t-distribution is used. Otherwise, the normal (z) distribution is used. See Help to Learn More							

c. Trend Analysis for OSCC Cases in Old Patients (>45 years old)

1. Graph

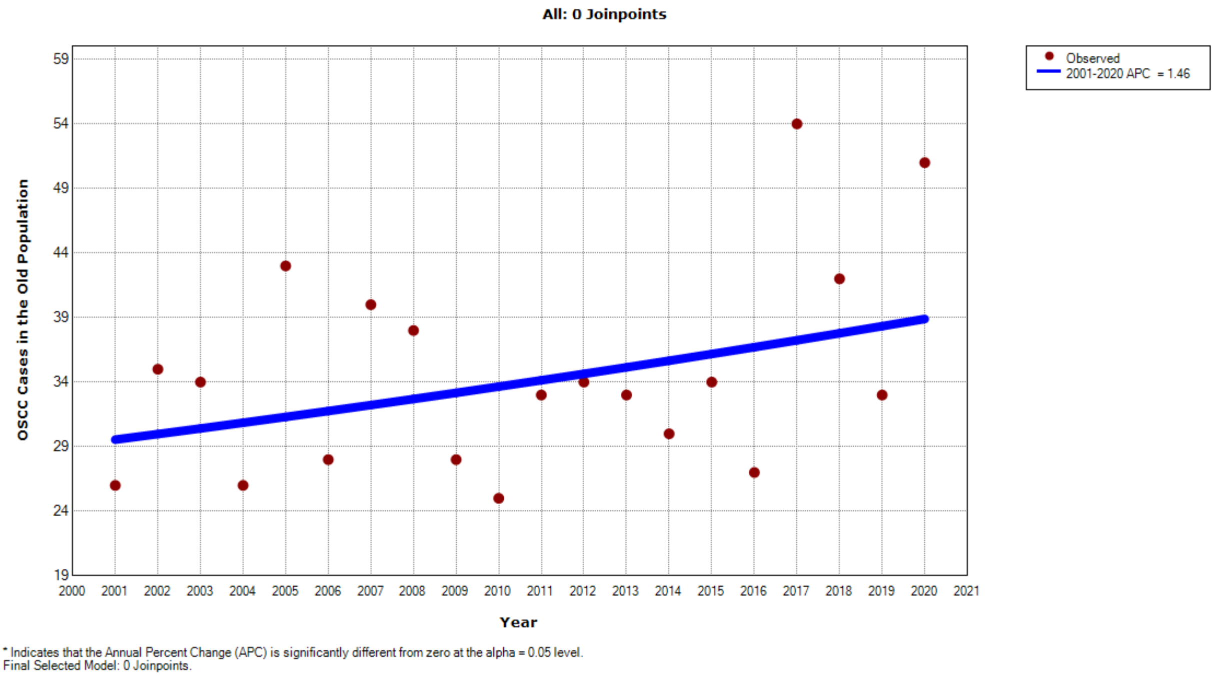

Figure 3. Trend analysis of OSCC cases in older Indonesian adults (>45 years old) from 2001 to 2020. The plotted lines represent annual percentage changes (APCs). *Significant change in APC compared to 0 ($p<0.05$) using the permutation model of logarithmically transformed data in Joinpoint regression analysis.

2. The significance test results obtained through the Monte Carlo permutation statistical method were used to identify the best-fitted line segment(s) in the Joinpoint regression analysis, representing significant trend changes (referred to as APC value) in the time series.

Annual Percent Change (APC)							
Segment	Lower Endpoint	Upper Endpoint	APC	Lower CI	Upper CI	Test Statistic (t)	Prob > |t|
1	2001.00	2020.00	1.46	-0.23	3.17	1.81	0.087
* Indicates that the Annual Percent Change (APC) is significantly different from zero at the alpha = 0.05 level.							

Average Annual Percent Change (AAPC)							
Range	Lower Endpoint	Upper Endpoint	AAPC	Lower CI	Upper CI	Test Statistic~	P-Value~
Full Range	2001.00	2020.00	1.46	-0.23	3.17	1.81	0.087
* Indicates that the AAPC is significantly different from zero at the alpha = 0.05 level.<br>~ If the AAPC is within one segment, the t-distribution is used. Otherwise, the normal (z) distribution is used. See Help to Learn More							

d. Trend Analysis for OSCC Cases in Males Patients

1. Graph

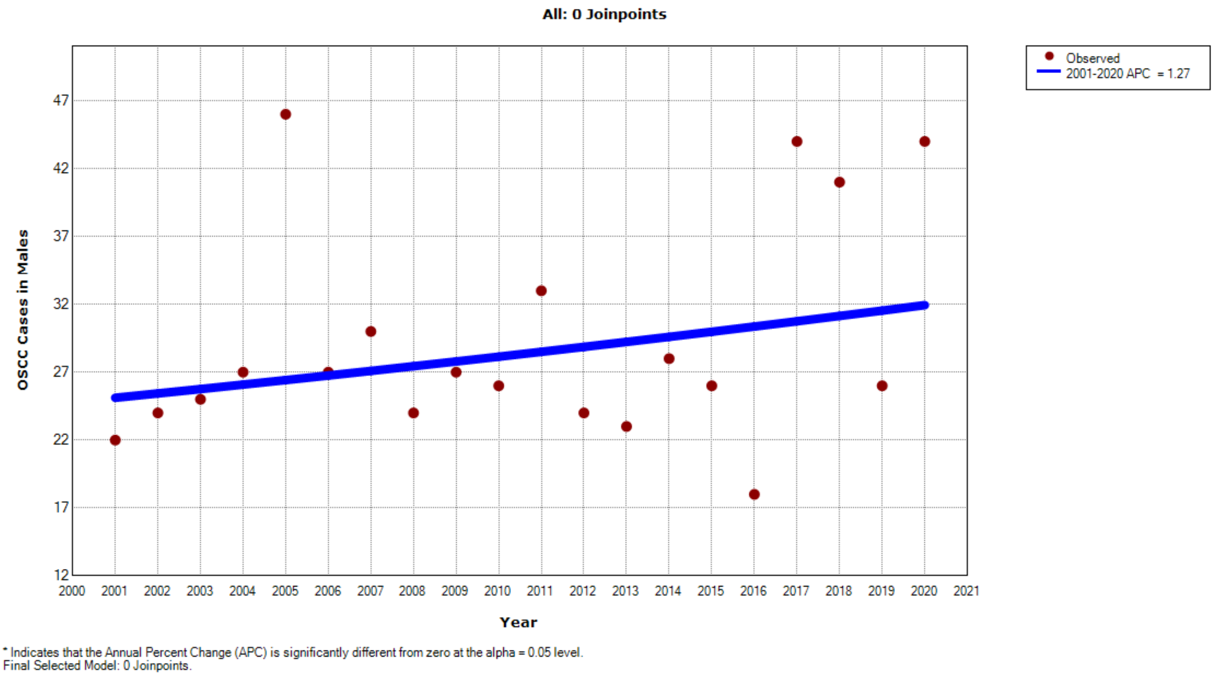

Figure 4. Trend analysis of OSCC cases in Indonesian males from 2001 to 2020. The plotted lines represent annual percentage changes (APCs). *Significant change in APC compared to 0 ($p<0.05$) using the permutation model of logarithmically transformed data in Joinpoint regression analysis.

2. The significance test results obtained through the Monte Carlo permutation statistical method were used to identify the best-fitted line segment(s) in the Joinpoint regression analysis, representing significant trend changes (referred to as APC value) in the time series.

Annual Percent Change (APC)							
Segment	Lower Endpoint	Upper Endpoint	APC	Lower CI	Upper CI	Test Statistic (t)	Prob > |t|
1	2001.00	2020.00	1.27	-0.76	3.35	1.31	0.207
* Indicates that the Annual Percent Change (APC) is significantly different from zero at the alpha = 0.05 level.							
**Average Annual Percent Change (AAPC)**							
Range	Lower Endpoint	Upper Endpoint	AAPC	Lower CI	Upper CI	Test Statistic~	P-Value~
Full Range	2001.00	2020.00	1.27	-0.76	3.35	1.31	0.207
* Indicates that the AAPC is significantly different from zero at the alpha = 0.05 level.<br>~ If the AAPC is within one segment, the t-distribution is used. Otherwise, the normal (z) distribution is used. See Help to Learn More							

e. Trend Analysis for OSCC Cases in Females Patients

1. Graph

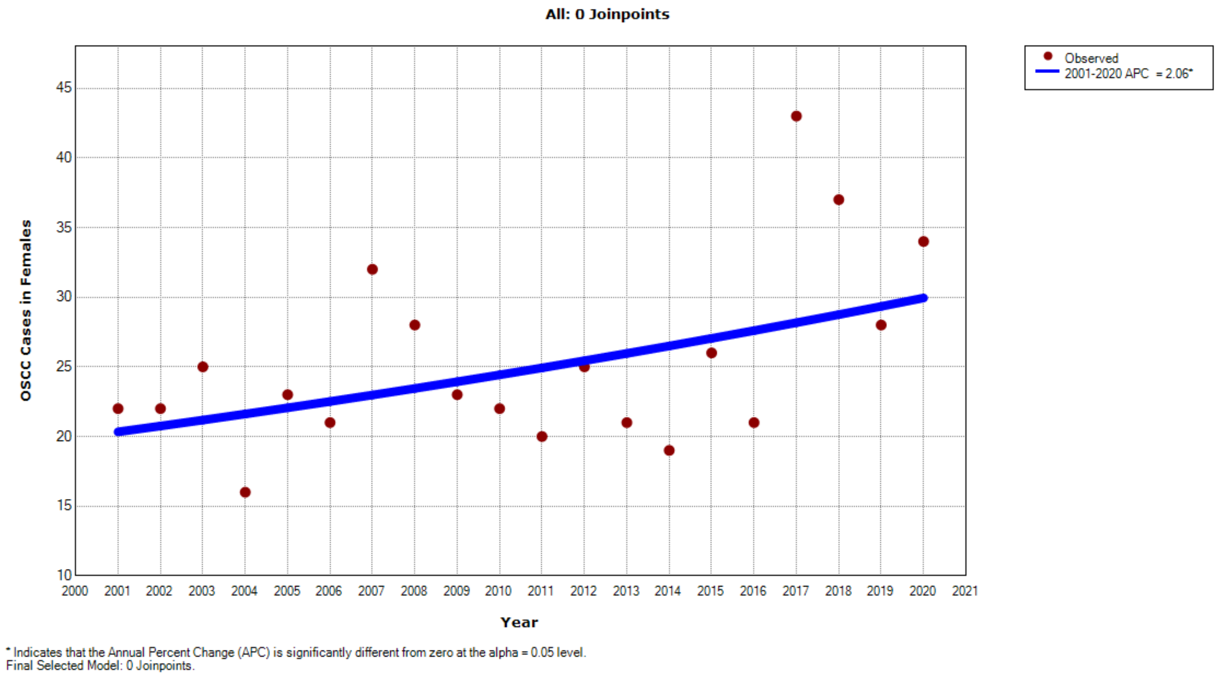

Figure 5. Trend analysis of OSCC cases in Indonesian females from 2001 to 2020. The plotted lines represent annual percentage changes (APCs). *Significant change in APC compared to 0 (p<0.05) using the permutation model of logarithmically transformed data in Joinpoint regression analysis.

2. The significance test results obtained through the Monte Carlo permutation statistical method were used to identify the best-fitted line segment(s) in the Joinpoint regression analysis, representing significant trend changes (referred to as APC value) in the time series.

Annual Percent Change (APC)							
Segment	Lower Endpoint	Upper Endpoint	APC	Lower CI	Upper CI	Test Statistic (t)	Prob > |t|
1	2001.00	2020.00	2.06*	0.29	3.86	2.45	0.025
* Indicates that the Annual Percent Change (APC) is significantly different from zero at the alpha = 0.05 level.							
**Average Annual Percent Change (AAPC)**							
**Range**	**Lower Endpoint**	**Upper Endpoint**	**AAPC**	**Lower CI**	**Upper CI**	**Test Statistic~**	**P-Value~**
Full Range	2001.00	2020.00	2.06*	0.29	3.86	2.45	0.025
* Indicates that the AAPC is significantly different from zero at the alpha = 0.05 level.<br>~ If the AAPC is within one segment, the t-distribution is used. Otherwise, the normal (z) distribution is used. See Help to Learn More							

f. Trend Analysis for OSCC Cases Originating from Tongue

1. Graph

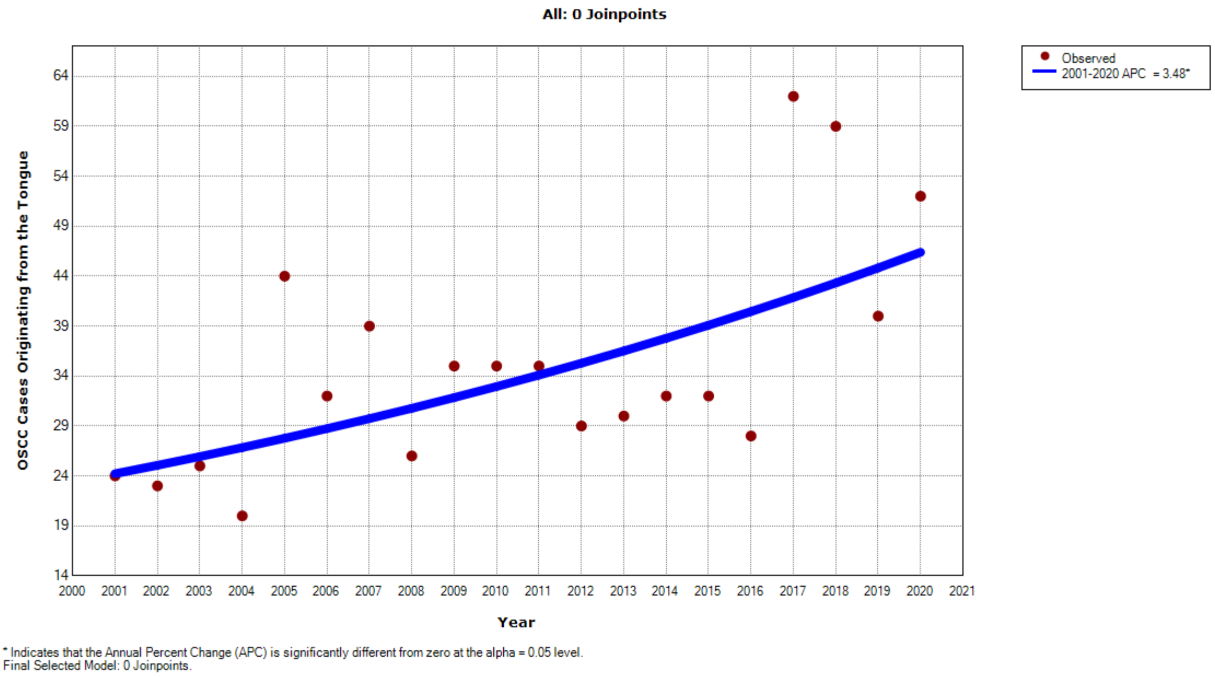

Figure 6. Trend analysis of Indonesian cases of OSCC originating from the tongue from 2001 to 2020. The plotted lines indicate annual percentage changes (APCs). *Significant change in APC compared to 0 (p<0.05) using the permutation model of logarithmically transformed data in Joinpoint regression analysis.

2. The significance test results obtained through the Monte Carlo permutation statistical method were used to identify the best-fitted line segment(s) in the Joinpoint regression analysis, representing significant trend changes (referred to as APC value) in the time series.

Annual Percent Change (APC)

Segment	Lower Endpoint	Upper Endpoint	APC	Lower CI	Upper CI	Test Statistic (t)	Prob > |t|
1	2001.00	2020.00	3.48*	1.52	5.47	3.76	0.001

* Indicates that the Annual Percent Change (APC) is significantly different from zero at the alpha = 0.05 level.

Average Annual Percent Change (AAPC)

Range	Lower Endpoint	Upper Endpoint	AAPC	Lower CI	Upper CI	Test Statistic~	P-Value~
Full Range	2001.00	2020.00	3.48*	1.52	5.47	3.76	0.001

* Indicates that the AAPC is significantly different from zero at the alpha = 0.05 level.
~ If the AAPC is within one segment, the t-distribution is used. Otherwise, the normal (z) distribution is used. See Help to Learn More

g. Trend Analysis for OSCC Cases Originating from Mouth NOS

1. Graph

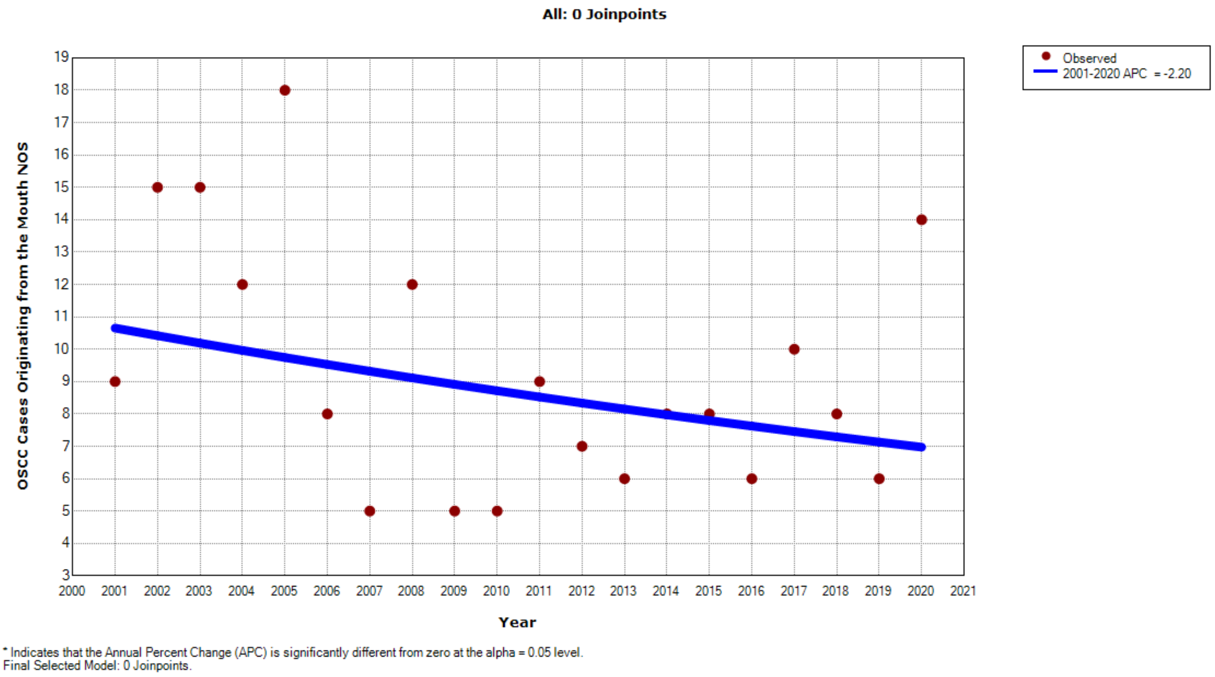

Figure 7. Trend analysis of Indonesian cases of OSCC originating from the mouth (not otherwise specified) from 2001 to 2020. The plotted lines indicate annual percentage changes (APCs). *Significant change in APC compared to 0 ($p<0.05$) using the permutation model of logarithmically transformed data in Joinpoint regression analysis.

2. The significance test results obtained through the Monte Carlo permutation statistical method were used to identify the best-fitted line segment(s) in the Joinpoint regression analysis, representing significant trend changes (referred to as APC value) in the time series.

Annual Percent Change (APC)

Segment	Lower Endpoint	Upper Endpoint	APC	Lower CI	Upper CI	Test Statistic (t)	Prob > |t|
1	2001.00	2020.00	-2.20	-5.22	0.90	-1.50	0.152

* Indicates that the Annual Percent Change (APC) is significantly different from zero at the alpha = 0.05 level.

Average Annual Percent Change (AAPC)

Range	Lower Endpoint	Upper Endpoint	AAPC	Lower CI	Upper CI	Test Statistic~	P-Value~
Full Range	2001.00	2020.00	-2.20	-5.22	0.90	-1.50	0.152

* Indicates that the AAPC is significantly different from zero at the alpha = 0.05 level.
~ If the AAPC is within one segment, the t-distribution is used. Otherwise, the normal (z) distribution is used. See Help to Learn More

h. Trend Analysis for OSCC Cases Originating from Palate

1. Graph

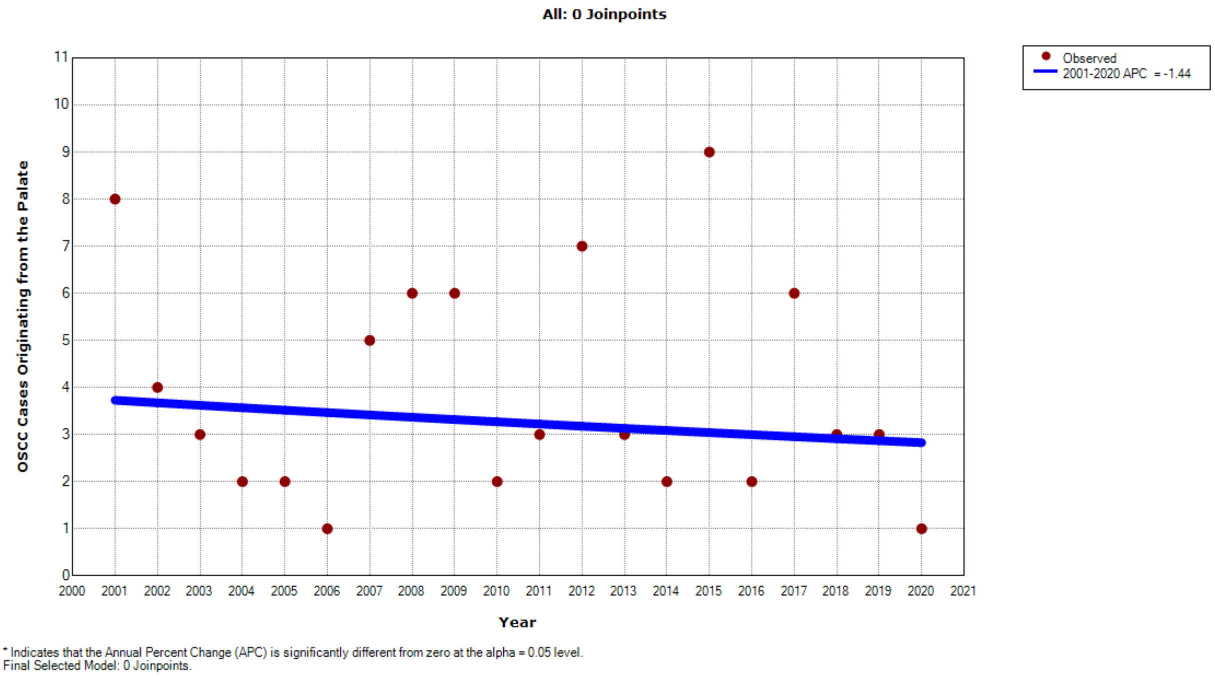

Figure 8. Trend analysis of Indonesian cases of OSCC originating from the palate from 2001 to 2020. The plotted lines indicate annual percentage changes (APCs). *Significant change in APC compared to 0 ($p<0.05$) using the permutation model of logarithmically transformed data in Joinpoint regression analysis.

2. The significance test results obtained through the Monte Carlo permutation statistical method were used to identify the best-fitted line segment(s) in the Joinpoint regression analysis, representing significant trend changes (referred to as APC value) in the time series.

Annual Percent Change (APC)							
Segment	Lower Endpoint	Upper Endpoint	APC	Lower CI	Upper CI	Test Statistic (t)	Prob > |t|
1	2001.00	2020.00	-1.44	-6.54	3.93	-0.58	0.572
* Indicates that the Annual Percent Change (APC) is significantly different from zero at the alpha = 0.05 level.							
**Average Annual Percent Change (AAPC)**							
Range	Lower Endpoint	Upper Endpoint	AAPC	Lower CI	Upper CI	Test Statistic~	P-Value~
Full Range	2001.00	2020.00	-1.44	-6.54	3.93	-0.58	0.572
* Indicates that the AAPC is significantly different from zero at the alpha = 0.05 level.<br>~ If the AAPC is within one segment, the t-distribution is used. Otherwise, the normal (z) distribution is used. See Help to Learn More							

i. Trend Analysis for OSCC Cases Originating from Gingiva

1. Graph

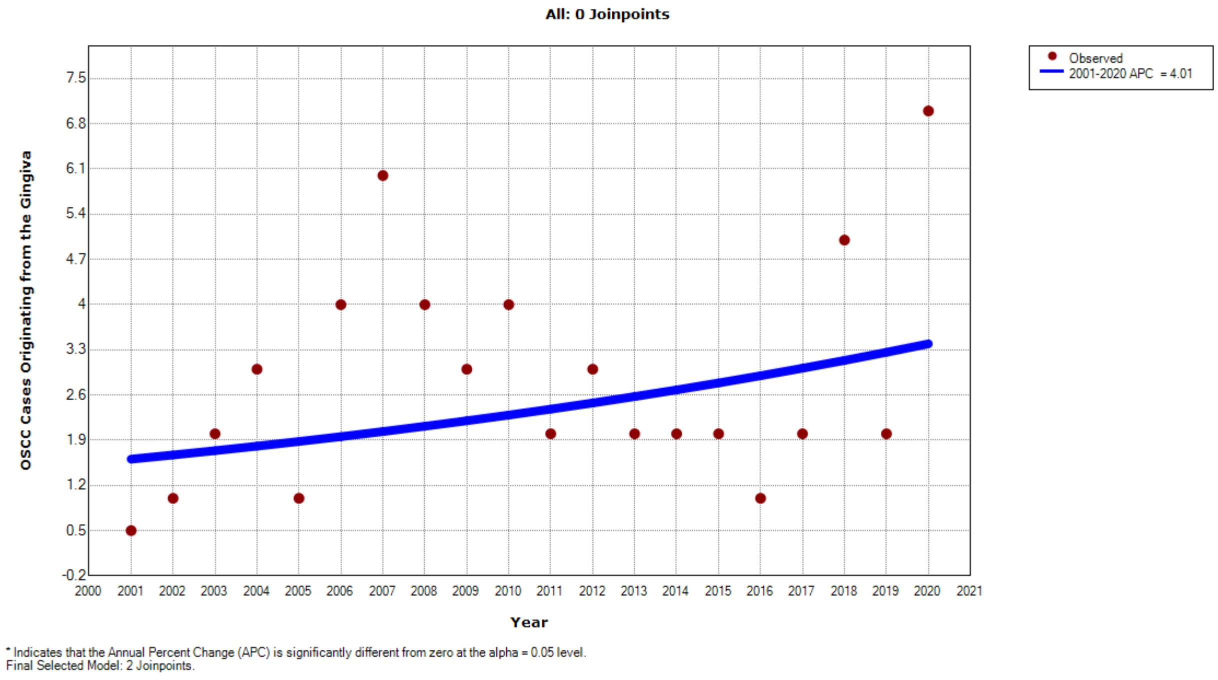

Figure 9. Trend analysis of Indonesian cases of OSCC originating from the gingiva during 2001–2020. Plotted lines indicate annual percentage changes (APCs). *Significant change in APC compared to 0 ($p<0.05$) using the permutation model of logarithmically transformed data in Joinpoint regression analysis.

2. The significance test results obtained through the Monte Carlo permutation statistical method were used to identify the best-fitted line segment(s) in the Joinpoint regression analysis, representing significant trend changes (referred to as APC value) in the time series.

Annual Percent Change (APC)							
Segment	Lower Endpoint	Upper Endpoint	APC	Lower CI	Upper CI	Test Statistic (t)	Prob > |t|
1	2001.00	2020.00	4.01	-1.28	9.59	1.58	0.131
* Indicates that the Annual Percent Change (APC) is significantly different from zero at the alpha = 0.05 level.							

Average Annual Percent Change (AAPC)							
Range	Lower Endpoint	Upper Endpoint	AAPC	Lower CI	Upper CI	Test Statistic~	P-Value~
Full Range	2001.00	2020.00	4.01	-1.28	9.59	1.58	0.131
* Indicates that the AAPC is significantly different from zero at the alpha = 0.05 level. ~ If the AAPC is within one segment, the t-distribution is used. Otherwise, the normal (z) distribution is used. See Help to Learn More							

j. Trend Analysis for OSCC Cases Originating from Lip

1. Graph

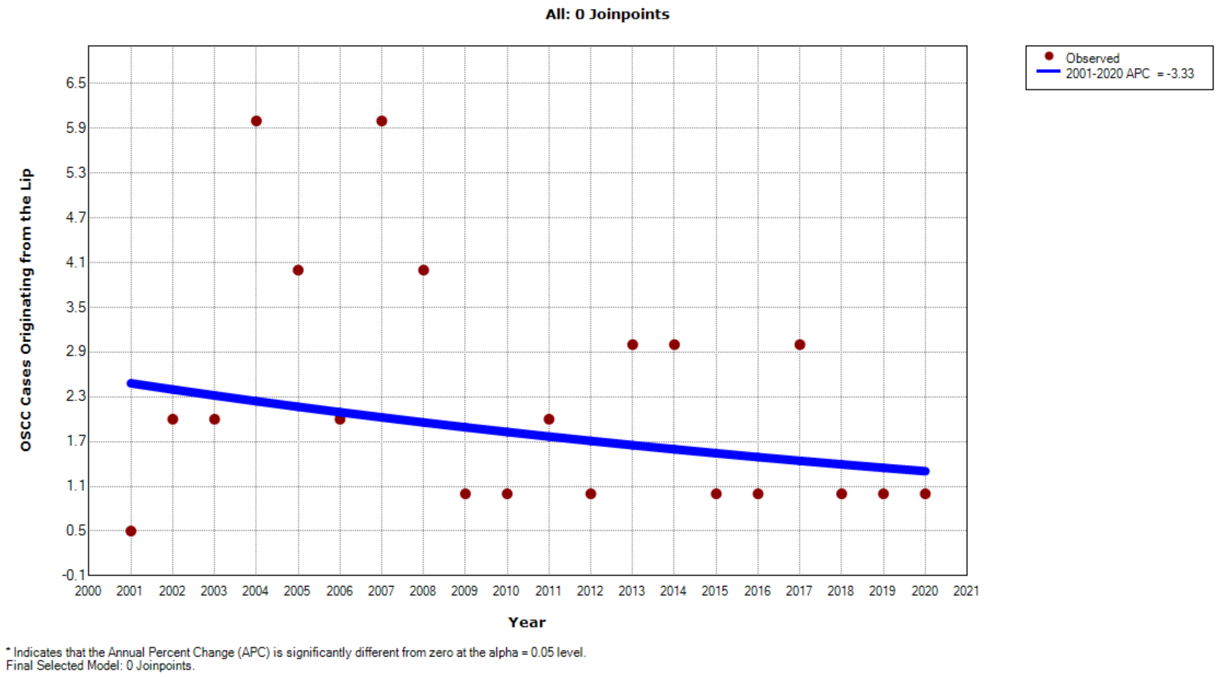

Figure 10. Trend analysis of Indonesian cases of OSCC originating from the lip during 2001–2020. Plotted lines indicate annual percentage changes (APCs). *Significant change in APC compared to 0 ($p<0.05$) using the permutation model of logarithmically transformed data in Joinpoint regression analysis.

2. The significance test results obtained through the Monte Carlo permutation statistical method were used to identify the best-fitted line segment(s) in the Joinpoint regression analysis, representing significant trend changes (referred to as APC value) in the time series.

Annual Percent Change (APC)							
Segment	Lower Endpoint	Upper Endpoint	APC	Lower CI	Upper CI	Test Statistic (t)	Prob > |t|
1	2001.00	2020.00	-3.33	-8.64	2.28	-1.26	0.223
* Indicates that the Annual Percent Change (APC) is significantly different from zero at the alpha = 0.05 level.							

Average Annual Percent Change (AAPC)							
Range	Lower Endpoint	Upper Endpoint	AAPC	Lower CI	Upper CI	Test Statistic~	P-Value~
Full Range	2001.00	2020.00	-3.33	-8.64	2.28	-1.26	0.223
* Indicates that the AAPC is significantly different from zero at the alpha = 0.05 level.<br>~ If the AAPC is within one segment, the t-distribution is used. Otherwise, the normal (z) distribution is used. See Help to Learn More							

k. Trend Analysis for OSCC Cases Originating from Buccal Mucosa

1. Graph

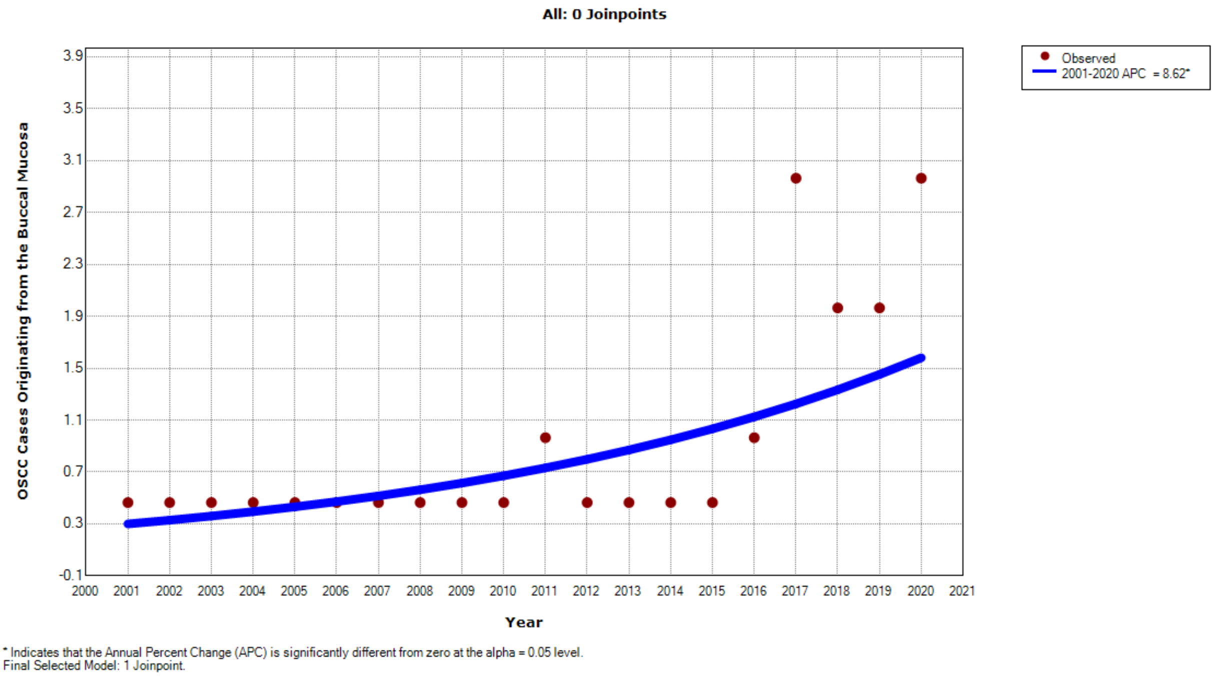

Figure 11. Trend analysis of Indonesian cases of OSCC originating from the buccal mucosa during 2001–2020. Plotted lines indicate annual percentage changes (APCs). *Significant change in APC compared to 0 ($p<0.05$) using the permutation model of logarithmically transformed data in Joinpoint regression analysis.

2. The significance test results obtained through the Monte Carlo permutation statistical method were used to identify the best-fitted line segment(s) in the Joinpoint regression analysis, representing significant trend changes (referred to as APC value) in the time series.

Annual Percent Change (APC)							
Segment	Lower Endpoint	Upper Endpoint	APC	Lower CI	Upper CI	Test Statistic (t)	Prob > |t|
1	2001.00	2020.00	8.62*	4.70	12.70	4.72	< 0.001
* Indicates that the Annual Percent Change (APC) is significantly different from zero at the alpha = 0.05 level.							

Average Annual Percent Change (AAPC)							
Range	Lower Endpoint	Upper Endpoint	AAPC	Lower CI	Upper CI	Test Statistic~	P-Value~
Full Range	2001.00	2020.00	8.62*	4.70	12.70	4.72	< 0.001
* Indicates that the AAPC is significantly different from zero at the alpha = 0.05 level. ~ If the AAPC is within one segment, the t-distribution is used. Otherwise, the normal (z) distribution is used. See Help to Learn More							

l. Trend Analysis for OSCC Cases Originating from the Floor of Mouth

1. Graph

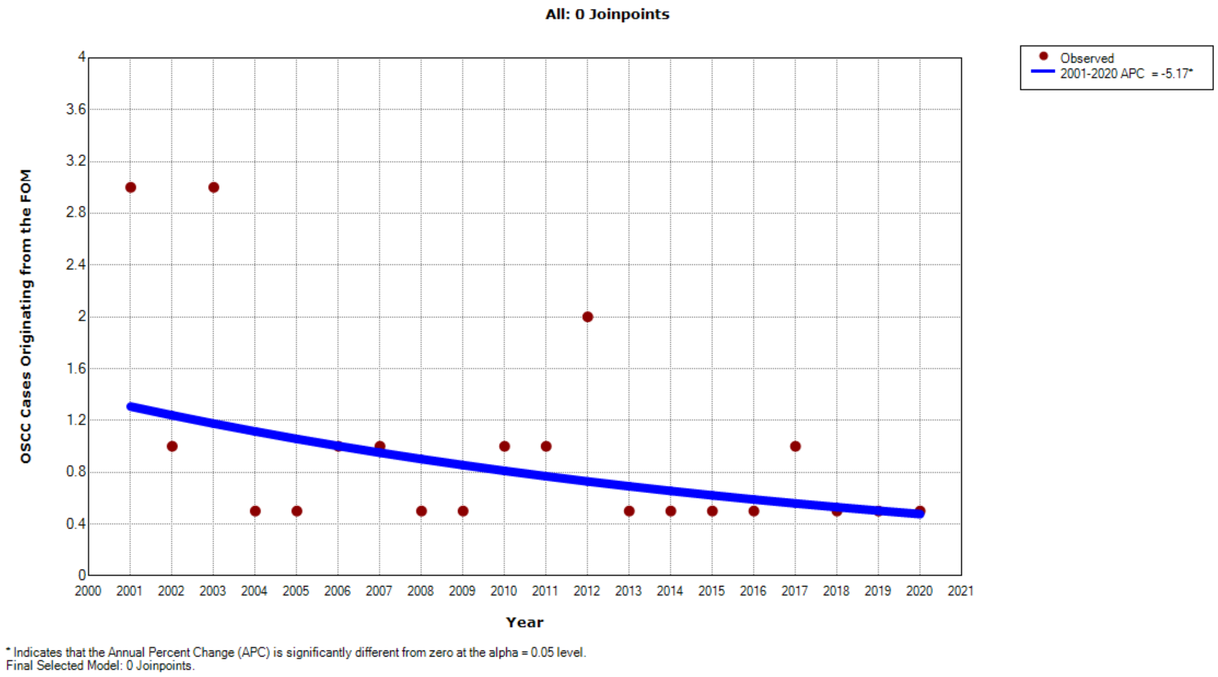

Figure 12. Trend analysis of Indonesian cases of OSCC originating from the floor of mouth (FOM) during 2001–2020. Plotted lines indicate annual percentage changes (APCs). *Significant change in APC compared to 0 ($p<0.05$) using the permutation model of logarithmically transformed data in Joinpoint regression analysis.

2. The significance test results obtained through the Monte Carlo permutation statistical method were used to identify the best-fitted line segment(s) in the Joinpoint regression analysis, representing significant trend changes (referred to as APC value) in the time series.

Annual Percent Change (APC)

Segment	Lower Endpoint	Upper Endpoint	APC	Lower CI	Upper CI	Test Statistic (t)	Prob > |t|
1	2001.00	2020.00	-5.17*	-9.23	-0.93	-2.55	0.020

* Indicates that the Annual Percent Change (APC) is significantly different from zero at the alpha = 0.05 level.

Average Annual Percent Change (AAPC)

Range	Lower Endpoint	Upper Endpoint	AAPC	Lower CI	Upper CI	Test Statistic~	P-Value~
Full Range	2001.00	2020.00	-5.17*	-9.23	-0.93	-2.55	0.020

* Indicates that the AAPC is significantly different from zero at the alpha = 0.05 level.
~ If the AAPC is within one segment, the t-distribution is used. Otherwise, the normal (z) distribution is used. See Help to Learn More
