# Supplementary material for: Estimated projection of oral squamous cell carcinoma annual incidence from twenty years registry data: a retrospective cross-sectional study in Indonesia
Source: PeerJ. 2023 Aug 29;11:e15911. doi: 10.7717/peerj.15911 (PMC10473041; doi:10.7717/peerj.15911)
Supplement: Supplemental Information 5 — In-depth analysis and validation of the generated model used to estimate the projection of oral squamous cell carcinoma (OSCC) cases over the next 5 years, employing linear/non-linear regression analysis. [file peerj-11-15911-s005.pdf]

Supplemental Data S3: Best-Fitted Model for Estimated Projection

In-depth analysis and validation of the generated model used to estimate the projection of oral squamous cell carcinoma (OSCC) cases over the next five years, employing linear/non-linear regression analysis.

a. Regression Model for Total OSCC Cases

1). Curve options

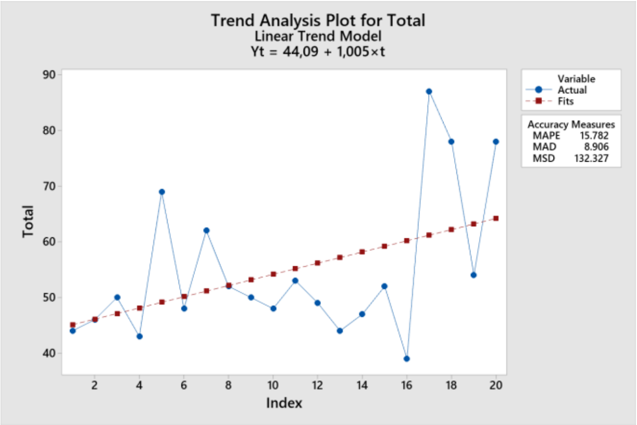

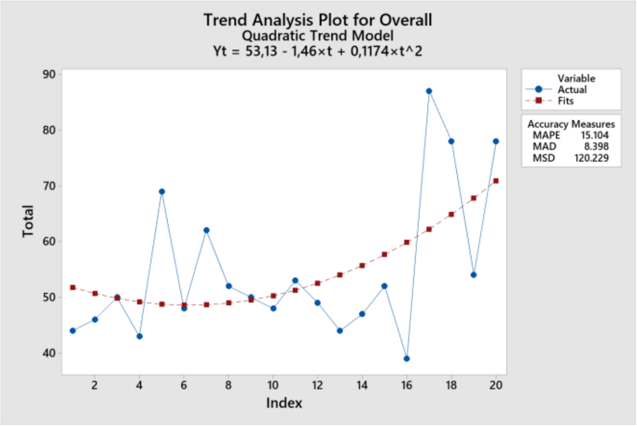

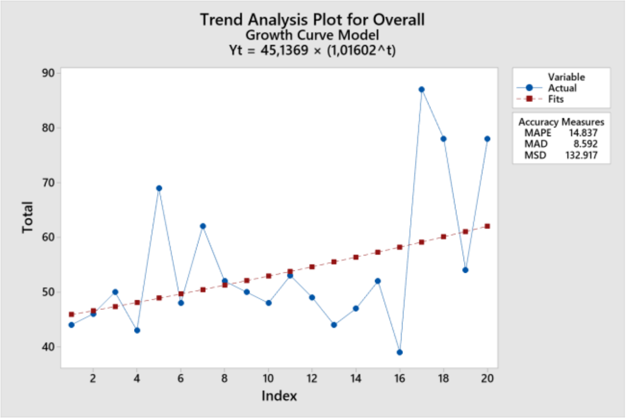

Model	Automatic Fitted-Curve	Accuracy Measurements		
		MAPE	MAD	MSD
Linear model	$Yt = 44.09 + 1.005t$	15.782	8.906	132.327
**Quadratic model***	**$Yt = 53.13 – 1.46t + 0.1174t^2$**	15.104	**8.398**	**120.229**
Growth curve model	$Yt = 45.1369 \times (1.01602^t)$	**14.837**	8.592	132.917

**The best-fitted model is the one that has the lowest value for three parameters (MAPE, MAD, and MSD), or at least for two parameters out of three, or at least having the lowest value for MAPE.*

2). Residual analysis, goodness-of-fit, and homoscedasticity for the Quadratic model

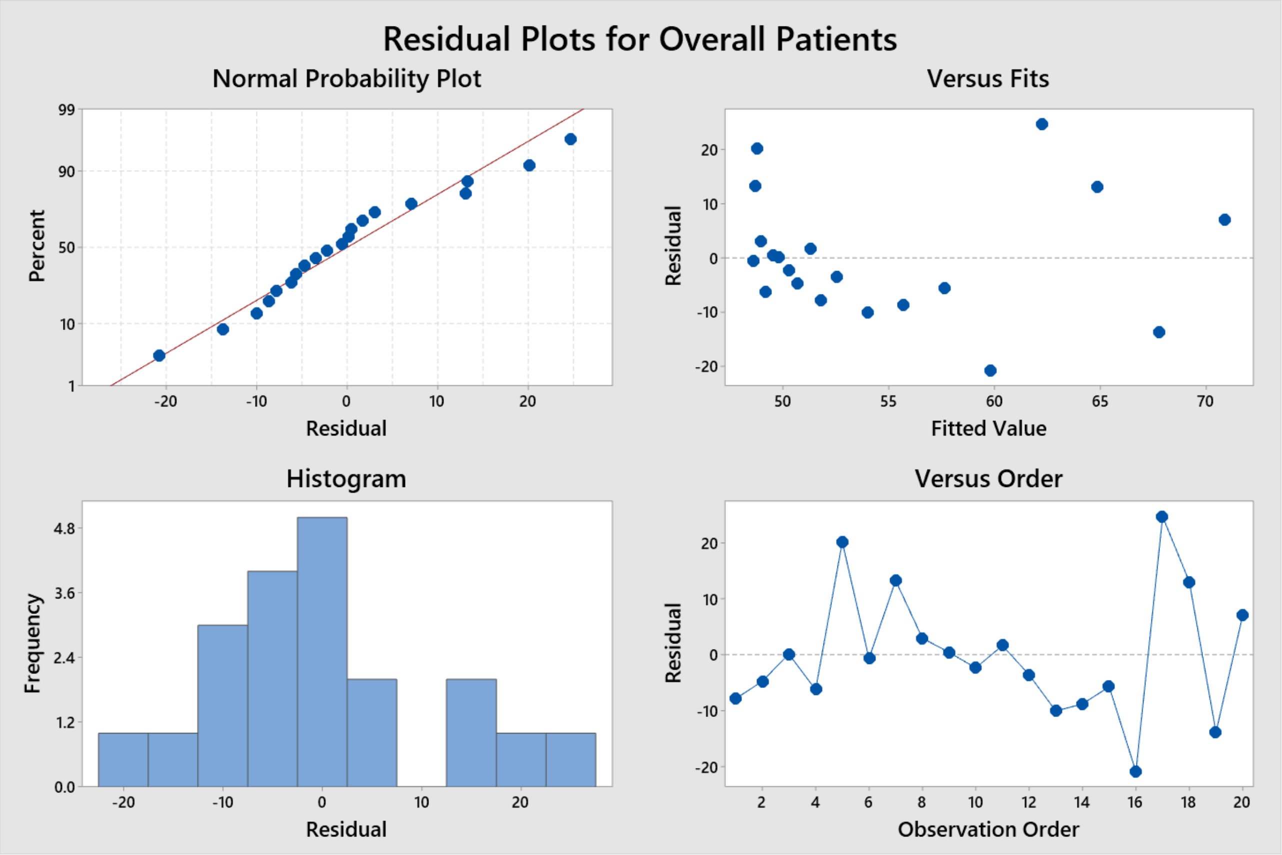

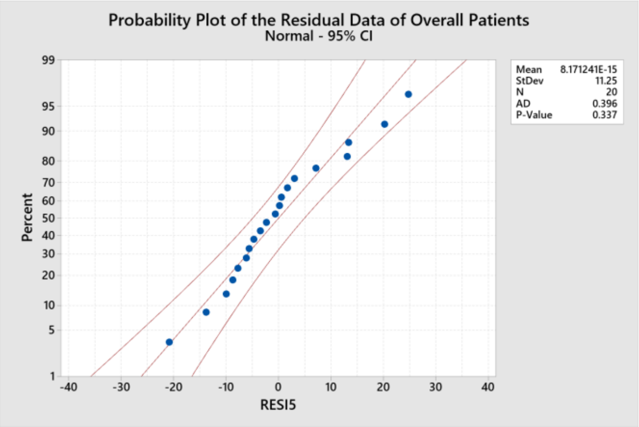

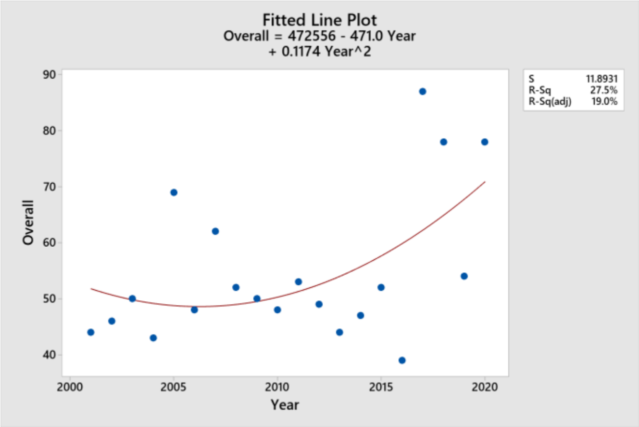

Interpretation:

- The normality test for the residuals yielded a p-value of **0.337**, suggesting a close alignment with the red line (“zero line” or “residuals mean line” or “identity line”) and indicating a normal distribution.
- The R-square value for the Quadratic model is **27.5%**, indicating a reasonable fit of the regression model to the data.
- The plot of fitted values versus residuals shows consistent variance across all fitted values, suggesting the assumption of homoscedasticity is likely satisfied.

3). Autocorrelataion test

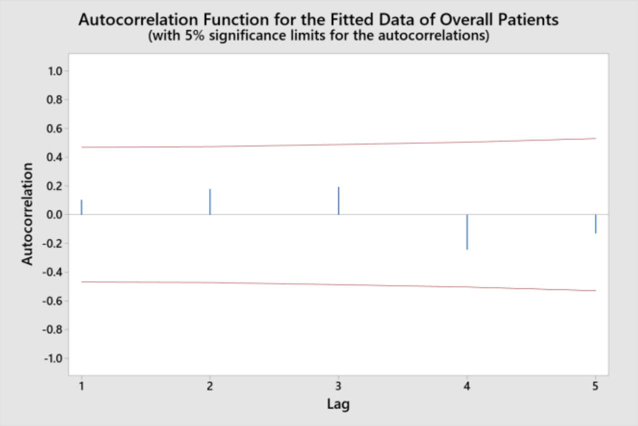

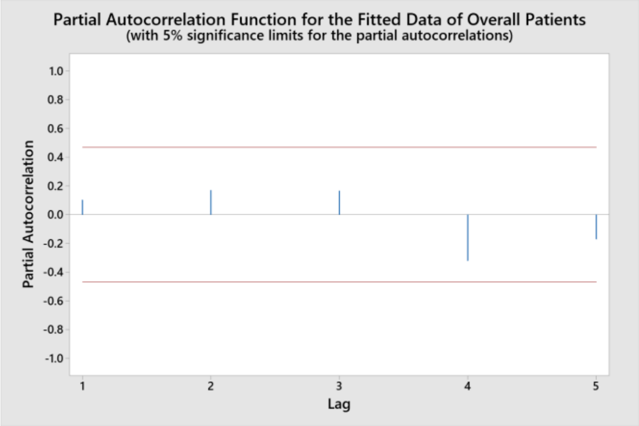

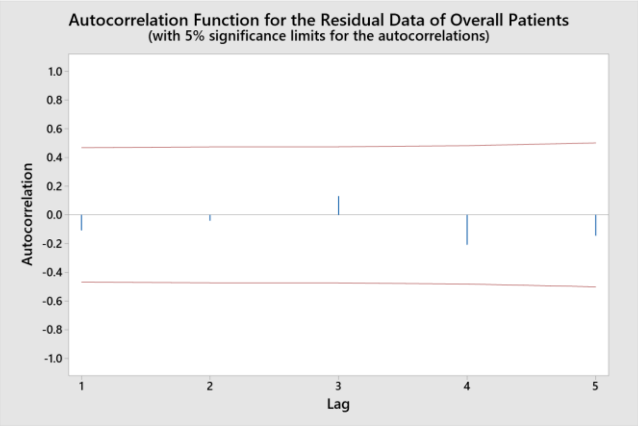

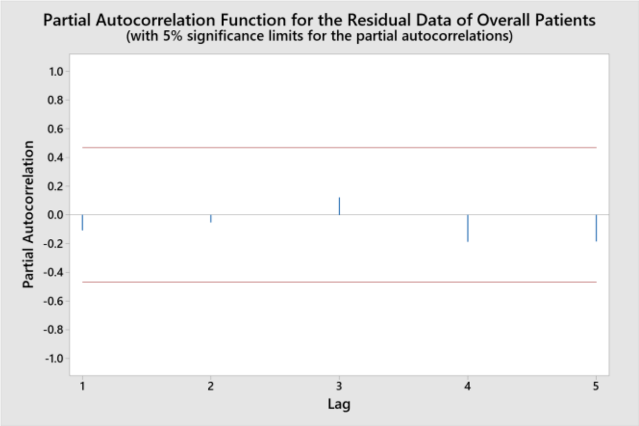

Interpretation:

The fitted and residual data of **overall patients** show a correlation **near zero**, indicating stationarity, independence, and absence of systematic patterns. This suggests that the model effectively captures underlying data patterns and produces accurate forecasts.

4). The results of the significance test for the slope of the Quadratic curve estimation

ANOVA

	Sum of Squares	df	Mean Square	F	Sig.
Regression	673.048	1	673.048	4.579	**0.046**
Residual	2645.502	18	146.972		
Total	3318.550	19			

The independent variable is the Year.

5). Forecasting the number of cases for the next five years using the Quadratic model

**Year**	**Forecasted Cases**
2021	74.2447
2022	77.8327
2023	81.6555
2024	85.7130
2025	90.0054
**Mean**	81.8903
**Total**	491.3416

b. Regression Model for OSCC Cases in Young Patients (≤45 years old)

1). Curve options

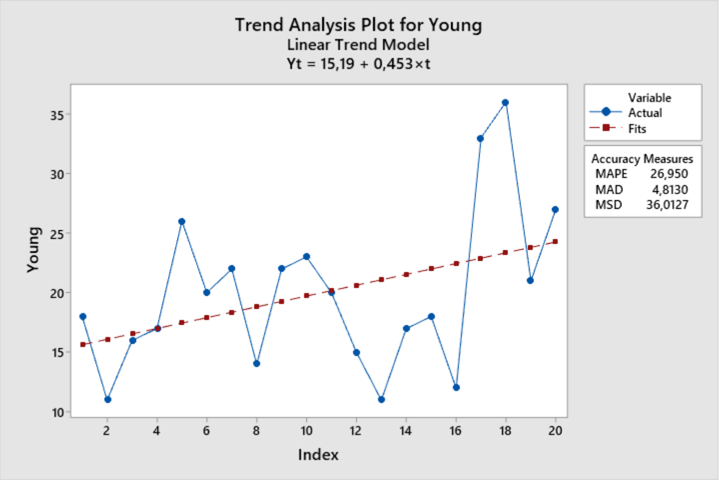

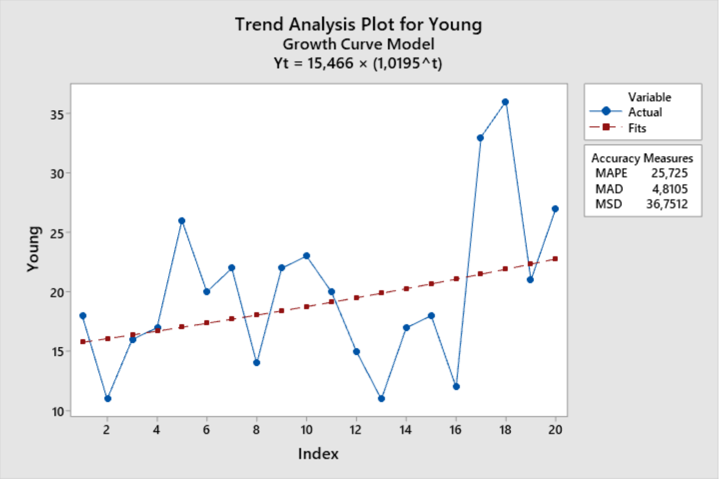

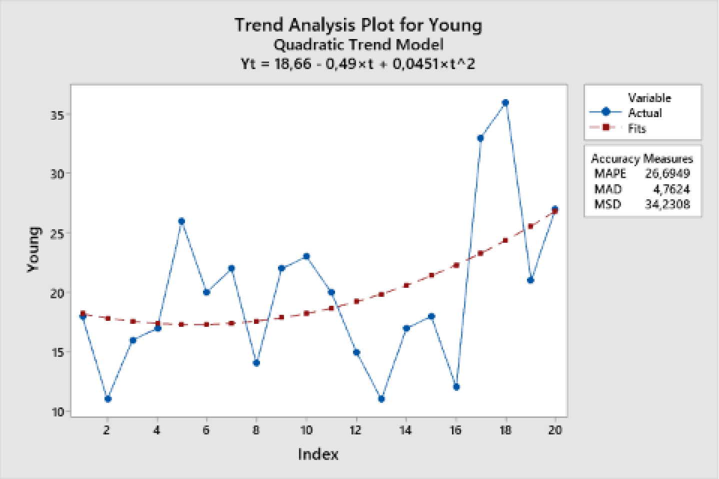

Model	Automatic Fitted-Curve	Accuracy Measurements		
		MAPE	MAD	MSD
Linear model	Yt = 15.19 + 0.453t	26.9506	4.8130	36.0127
**Quadratic model***	**Yt = 18.66 – 0.49t + 0.0451t^2**	**26.6949**	**4.7624**	**34.2308**
Growth curve	Yt = 15.466 × (1.0195^t)	25.7255	4.8105	36.7512

**The best-fitted model is the one that has the lowest value for three parameters (MAPE, MAD, and MSD), or at least for two parameters out of three, or at least having the lowest value for MAPE.*

2). Residual analysis, goodness-of-fit, and homoscedasticity for the Quadratic model

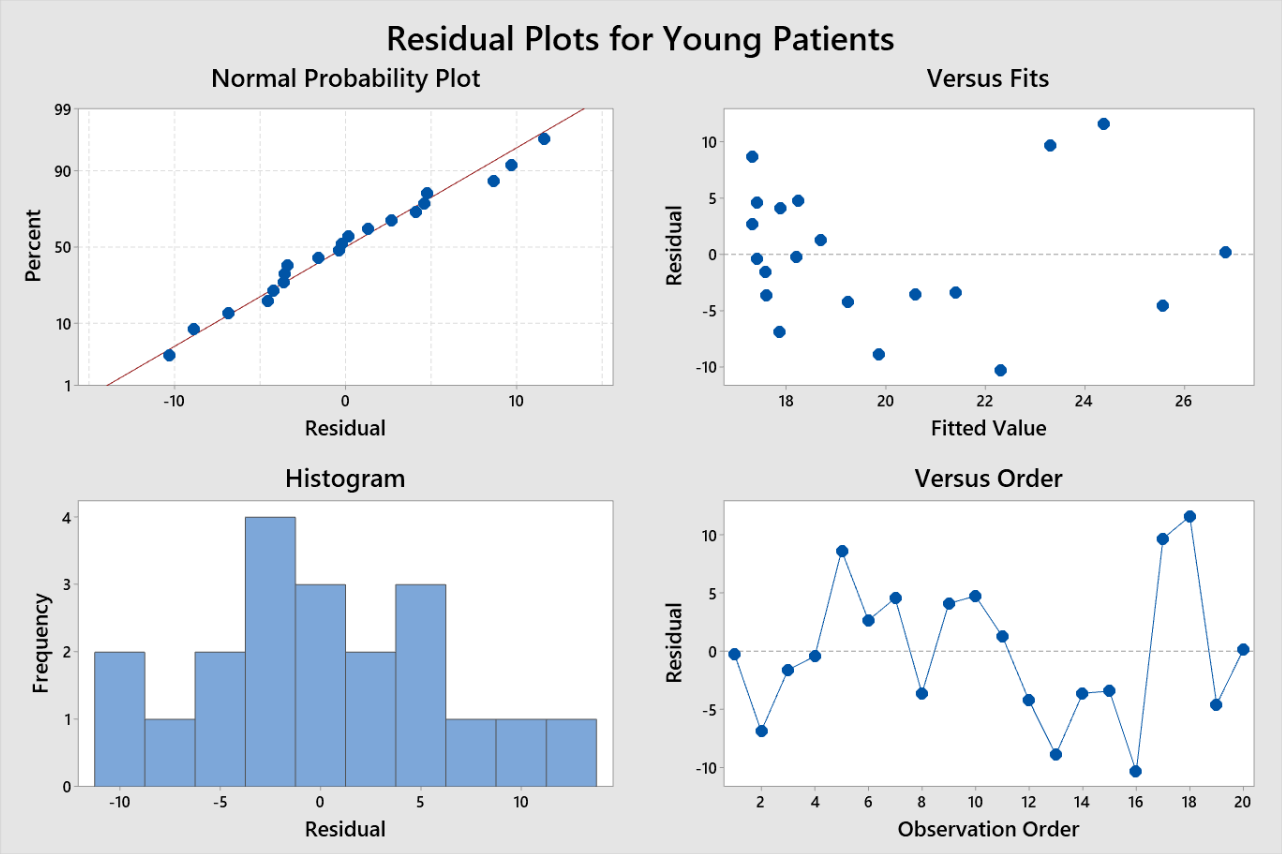

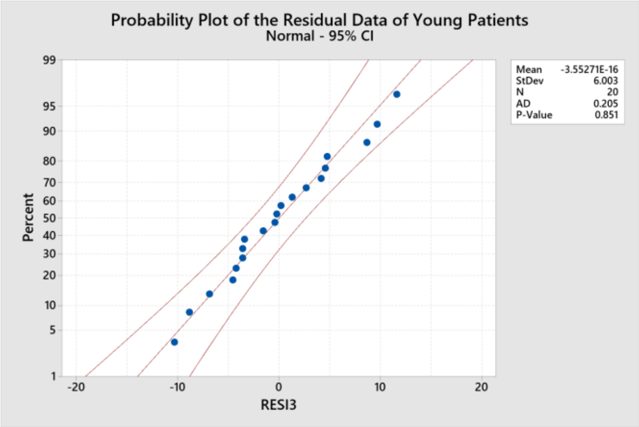

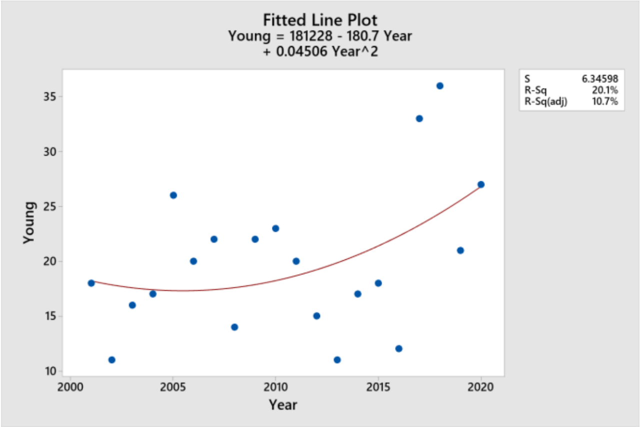

Interpretation:

- The normality test for the residuals yielded a p-value of **0.851**, suggesting a close alignment with the red line ("zero line" or "residuals mean line" or "identity line")and indicating a normal distribution.
- The R-square value for the Quadratic model is **20.1%**, indicating a reasonable fit of the regression model to the data.
- The plot of fitted values versus residuals shows consistent variance across all fitted values, suggesting the assumption of homoscedasticity is likely satisfied.

3). Autocorrelataion test

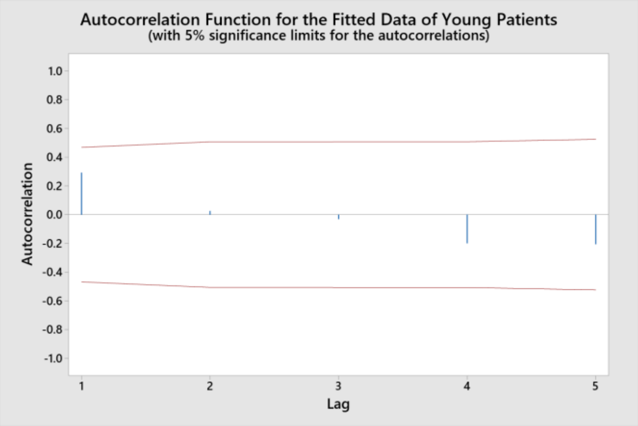

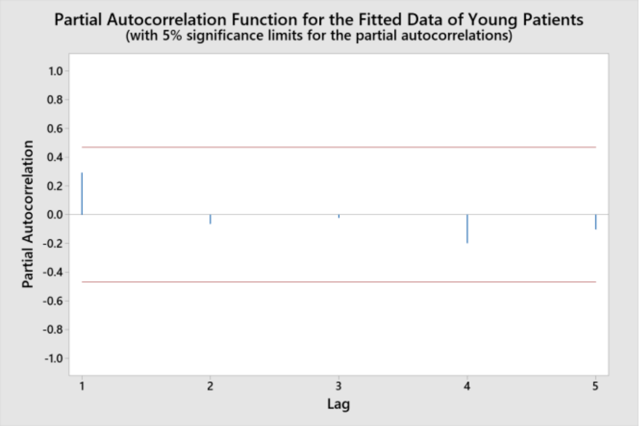

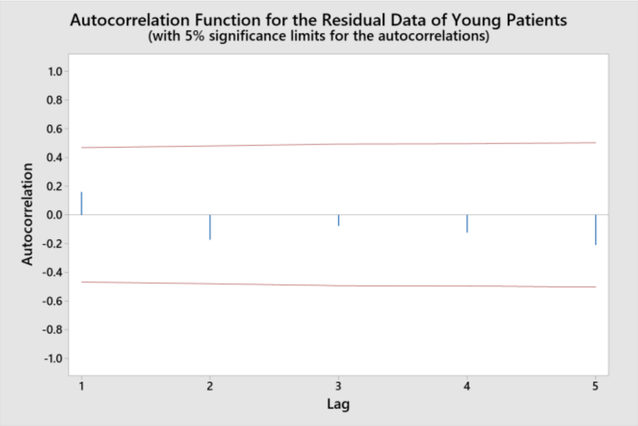

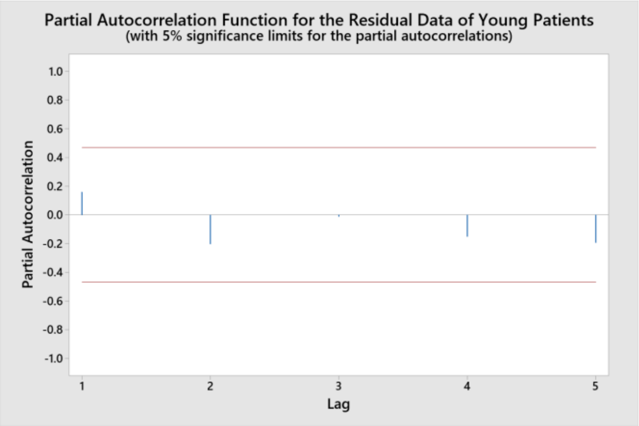

Interpretation:

The fitted and residual data of **young patients** show a correlation **near zero**, indicating stationarity, independence, and absence of systematic patterns. This suggests that the model effectively captures underlying data patterns and produces accurate forecasts.

4). The results of the significance test for the slope of the Quadratic curve estimation

ANOVA

	Sum of Squares	df	Mean Square	F	Sig.
Regression	136.873	1	136.873	3.421	0.081
Residual	720.077	18	40.004		
Total	856.950	19			

The independent variable is the Year.

5). Forecasting the number of cases for the next five years using the Quadratic model

Year	Forecasted Cases
2021	28.1798
2022	29.6244
2023	31.1592
2024	32.7840
2025	34.4989
**Mean**	31.2493
**Total**	187.4956

c. Regression Model for OSCC Cases in Old Patients (>45 years old)

1). Curve options

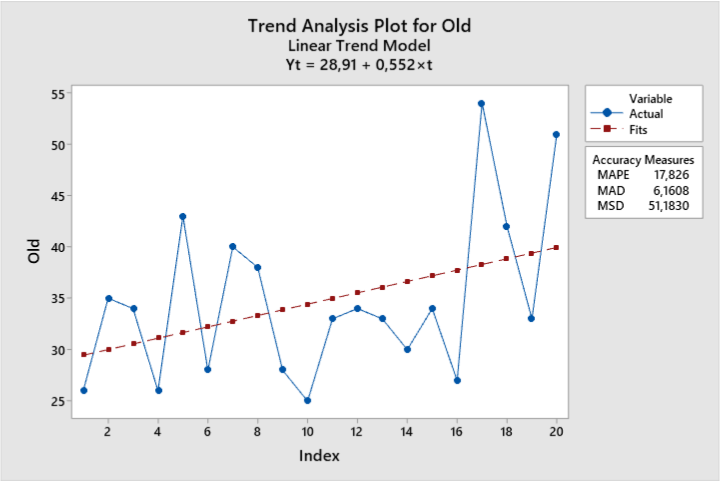

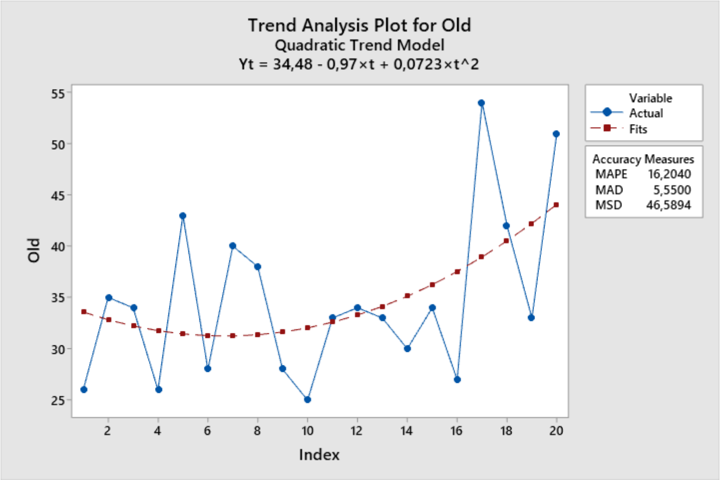

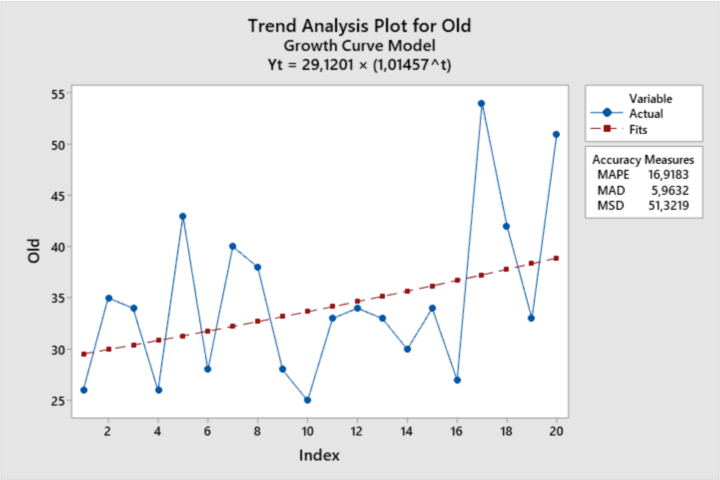

Model	Automatic Fitted-Curve	Accuracy Measurements		
		MAPE	MAD	MSD
Linear model	Yt = 28.91 + 0.552t	17.8266	6.1608	51.1830
**Quadratic model***	**Yt = 34.48 – 0.97t + 0.0723t^2**	**16.2040**	**5.5500**	**46.5894**
Growth curve model	Yt = 29.1201 × (1.01457t)	16.9183	5.9632	51.3219

**The best-fitted model is the one that has the lowest value for three parameters (MAPE, MAD, and MSD), or at least for two parameters out of three, or at least having the lowest value for MAPE.*

2). Residual analysis, goodness-of-fit, and homoscedasticity for the Quadratic model

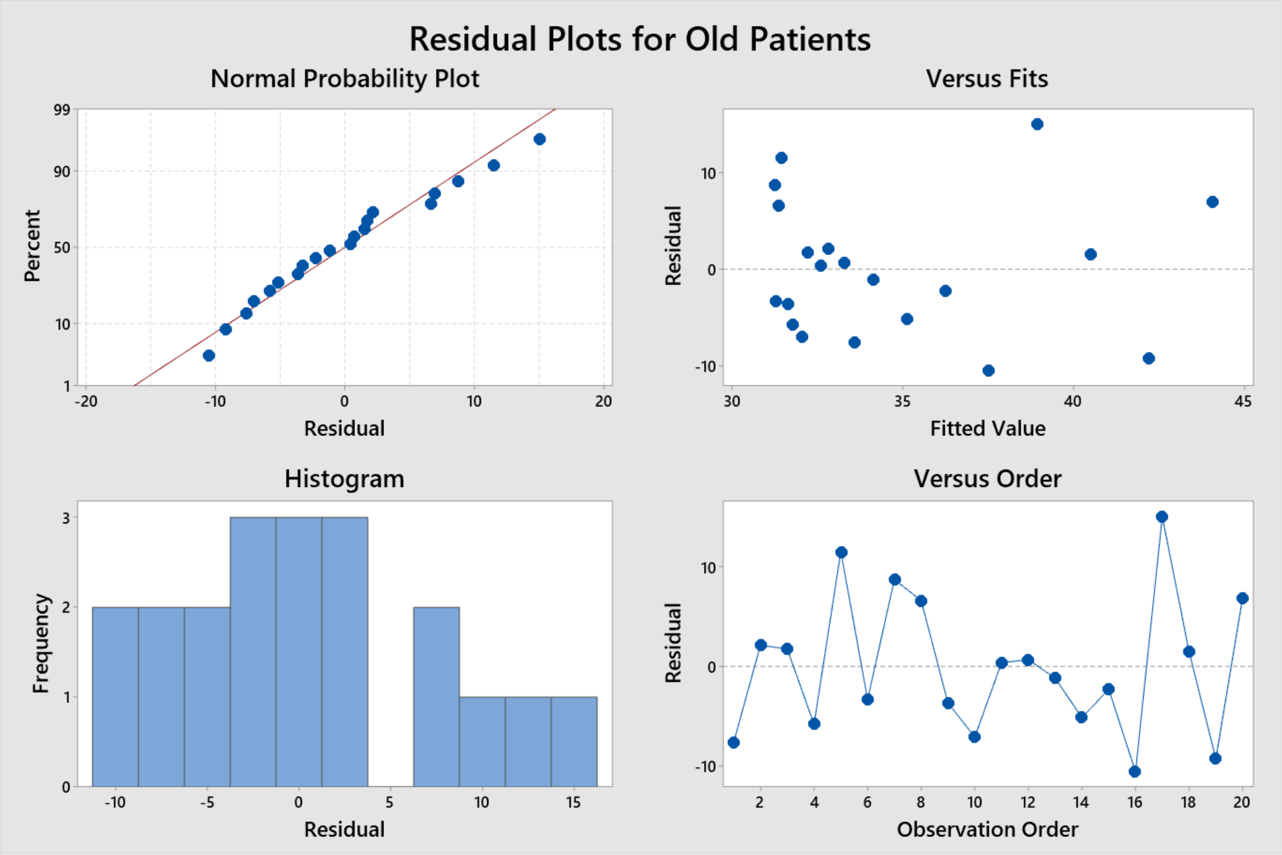

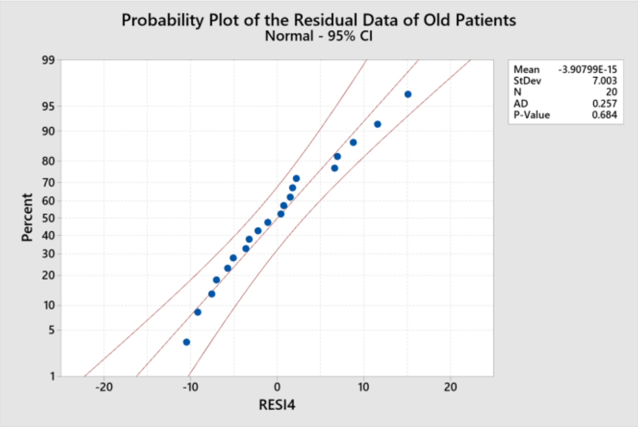

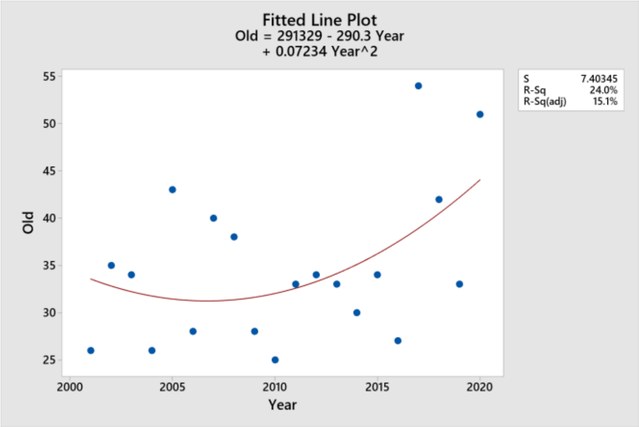

Interpretation:

- The normality test for the residuals yielded a p-value of **0.684**, suggesting a close alignment with the red line ("zero line" or "residuals mean line" or "identity line") and indicating a normal distribution.
- The R-square value for the Quadratic model is **24.0%**, indicating a reasonable fit of the regression model to the data.
- The plot of fitted values versus residuals shows consistent variance across all fitted values, suggesting the assumption of homoscedasticity is likely satisfied.

3). Autocorrelataion test

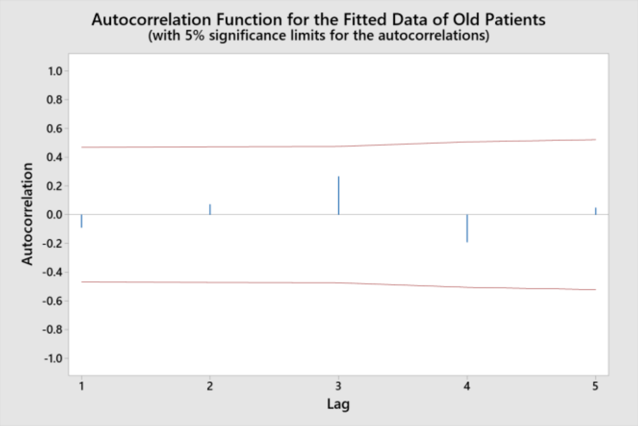

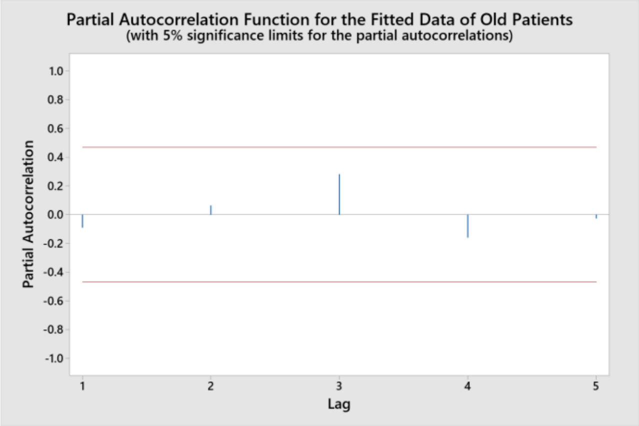

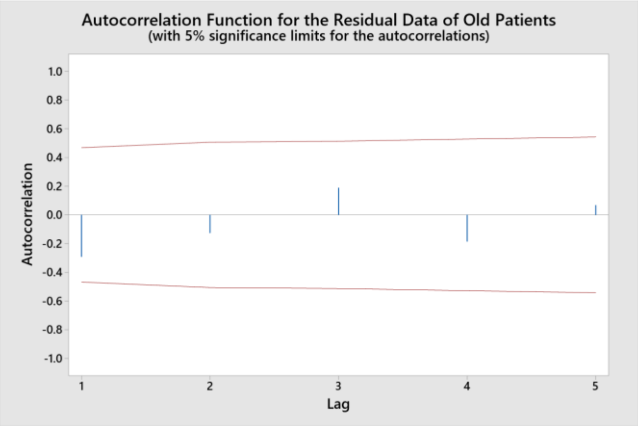

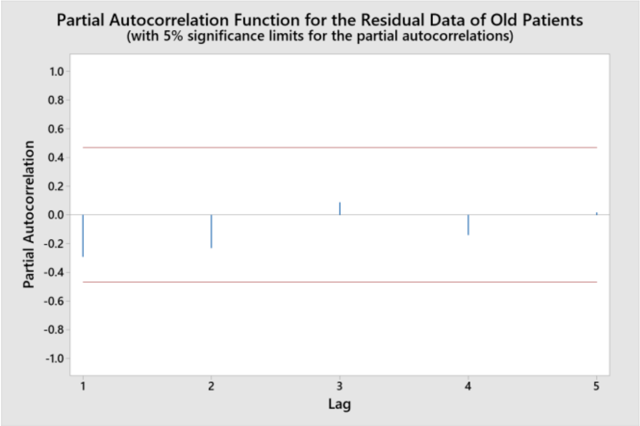

Interpretation:

The fitted and residual data of **old patients** show a correlation **near zero**, indicating stationarity, independence, and absence of systematic patterns. This suggests that the model effectively captures underlying data patterns and produces accurate forecasts.

4). The results of the significance test for the slope of the Quadratic curve estimation

ANOVA

	Sum of Squares	df	Mean Square	F	Sig.
Regression	202.888	1	202.888	3.569	0.075
Residual	1023.312	18	56.851		
Total	1226.200	19			

The independent variable is the Year.

5). Forecasting the number of cases for the next five years using the Quadratic model

Year	Forecasted Cases
2021	46.0649
2022	48.2083
2023	50.4963
2024	52.9290
2025	55.5064
**Mean**	50.6410
**Total**	303.8459

d. Regression Model for OSCC Cases in Male Patients

1). Curve options

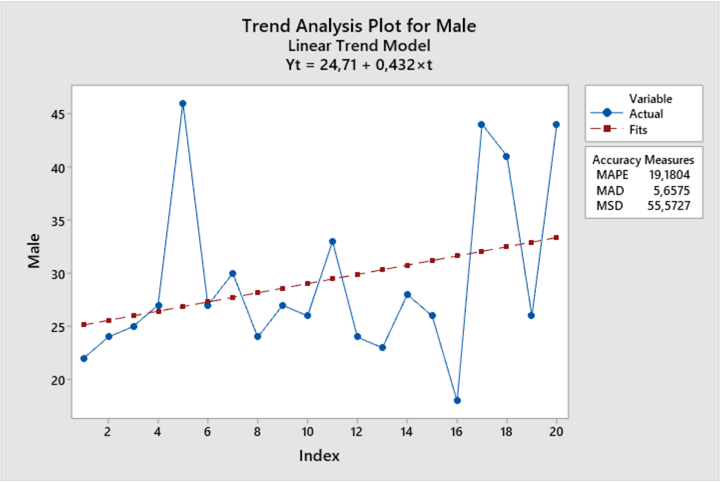

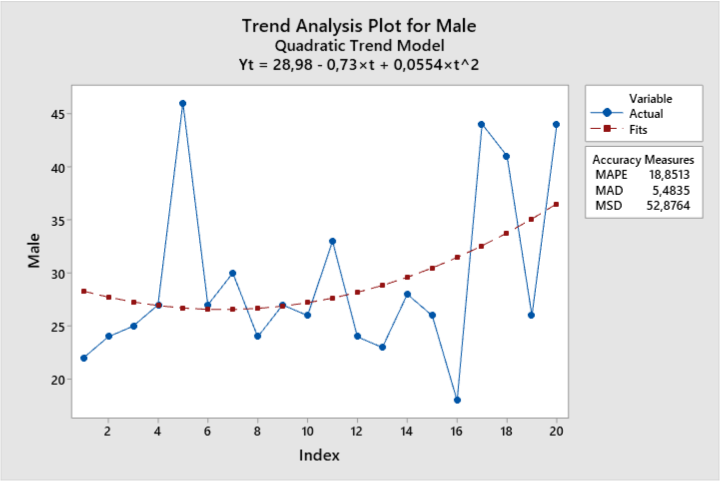

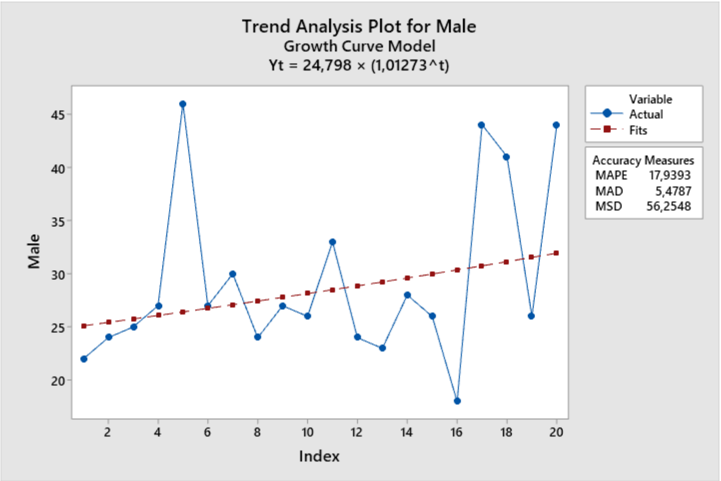

Model	Automatic Fitted-Curve	Accuracy Measurements		
		MAPE	MAD	MSD
Linear model	Yt = 24.71 + 0.432t	19.1804	5.6575	55.5727
**Quadratic model***	**$Yt = 28.98 – 0.73t + 0.0554t^2$**	**18.8513**	**5.4835**	**52.8764**
Growth curve model	$Yt = 24.798 \times (1.01273^t)$	17.9393	5.4787	56.2548

**The best-fitted model is the one that has the lowest value for three parameters (MAPE, MAD, and MSD), or at least for two parameters out of three, or at least having the lowest value for MAPE.*

2). Residual analysis, goodness-of-fit, and homoscedasticity for the Quadratic model

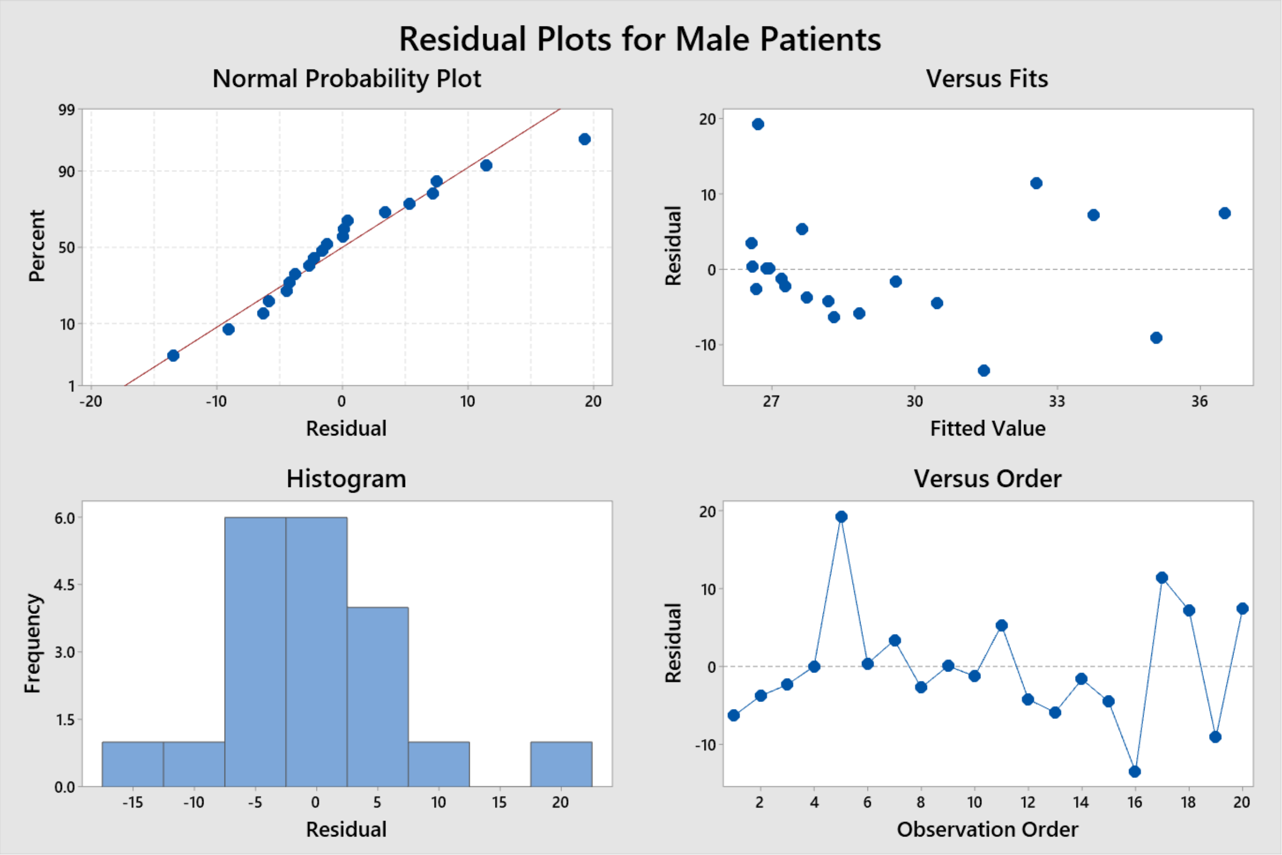

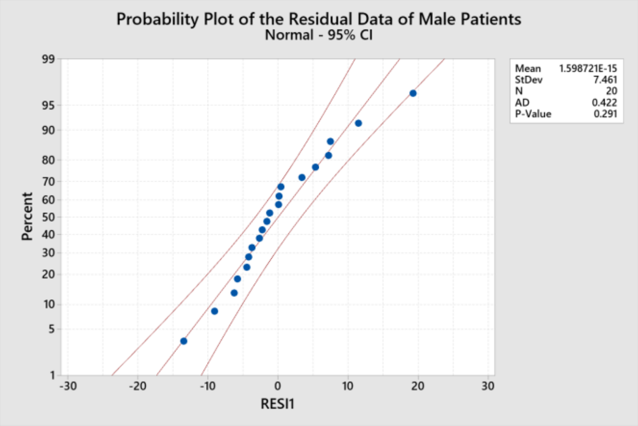

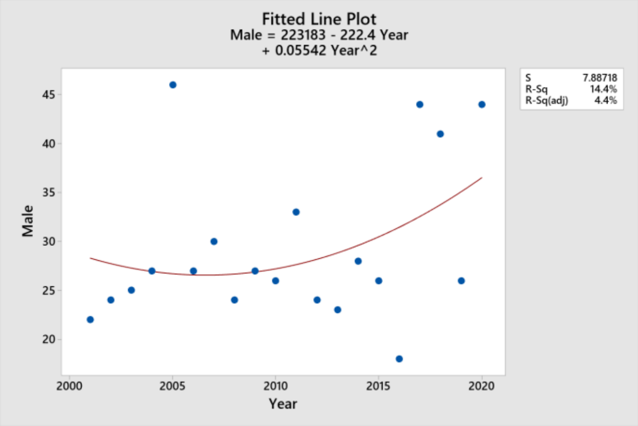

Interpretation:

- The normality test for the residuals yielded a p-value of **0.291**, suggesting a close alignment with the red line (“zero line” or “residuals mean line” or “identity line”) and indicating a normal distribution.
- The R-square value for the Quadratic model is **14.4%,** indicating a reasonable fit of the regression model to the data.
- The plot of fitted values versus residuals shows consistent variance across all fitted values, suggesting the assumption of homoscedasticity is likely satisfied.

3). Autocorrelataion test

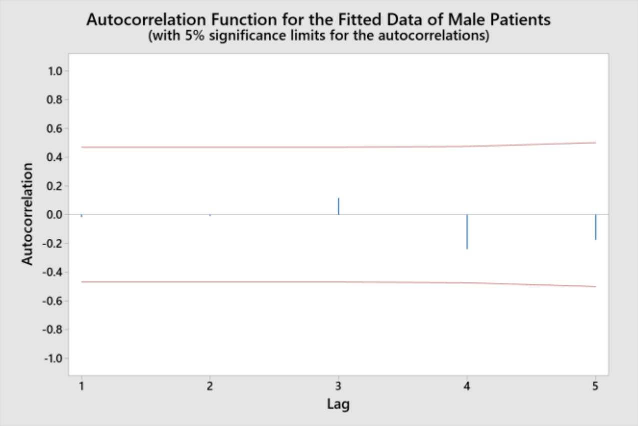

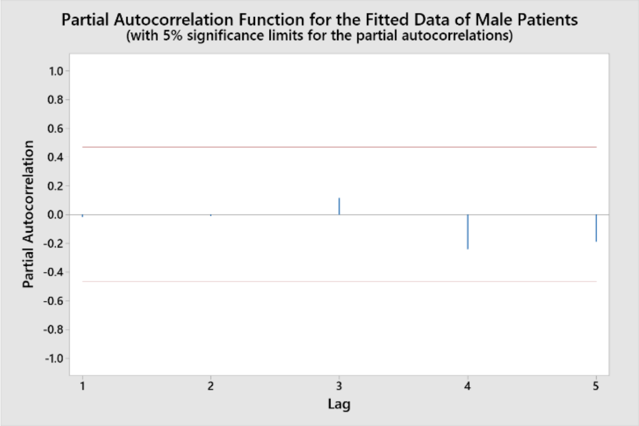

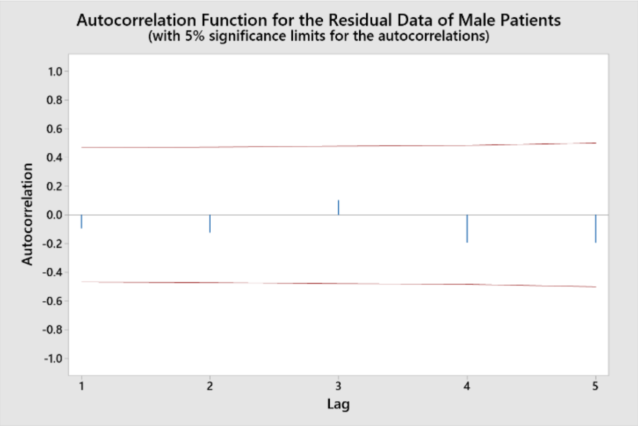

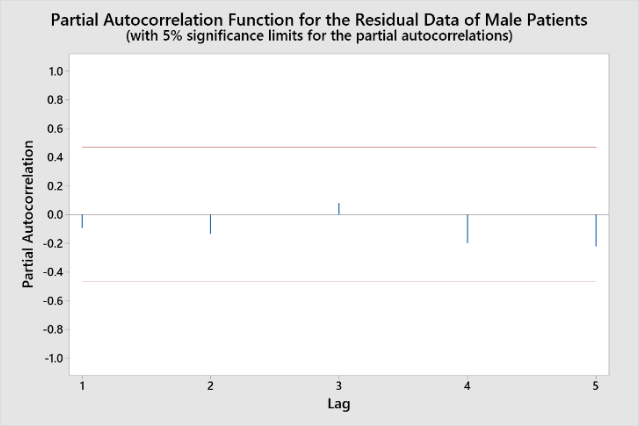

Interpretation:

The fitted and residual data of **male patients** show a correlation **near zero**, indicating stationarity, independence, and absence of systematic patterns. This suggests that the model effectively captures underlying data patterns and produces accurate forecasts.

4). The results of the significance test for the slope of the Quadratic curve estimation

ANOVA

	Sum of Squares	df	Mean Square	F	Sig.
Regression	124.504	1	124.504	2.017	0.173
Residual	1111.246	18	61.736		
Total	1235.750	19			

The independent variable is the Year.

5). Forecasting the number of cases for the next five years using the Quadratic model

Year	Forecasted Cases
2021	38.0570
2022	39.7086
2023	41.4711
2024	43.3444
2025	45.3286
**Mean**	41.5819
**Total**	249.4916

e. Regression Model for OSCC Cases in Female Patients

1). Curve options

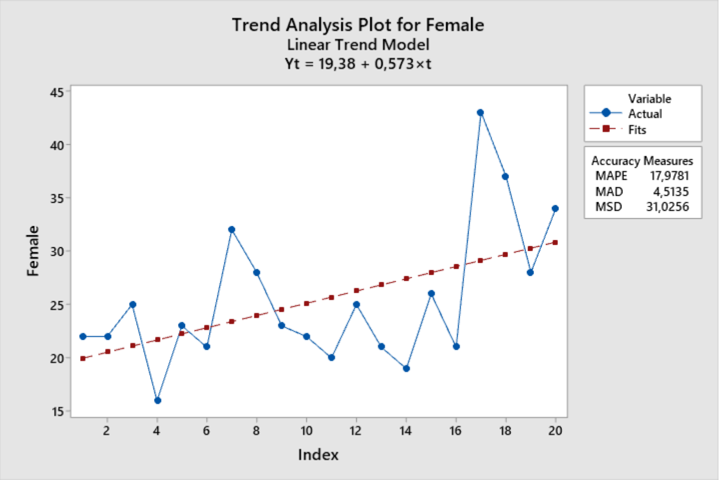

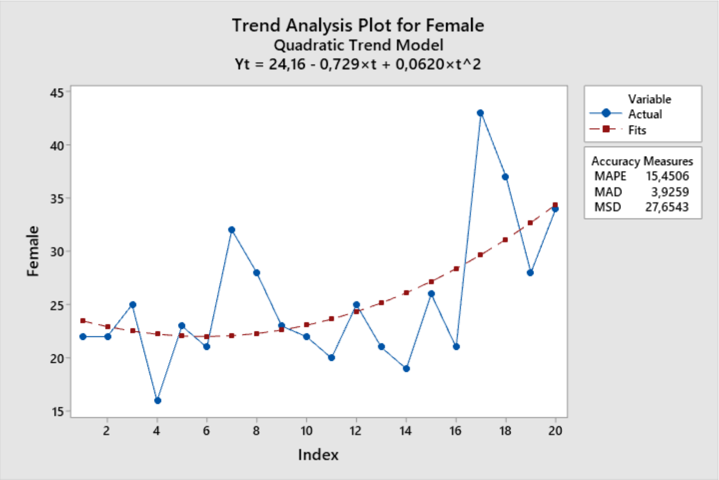

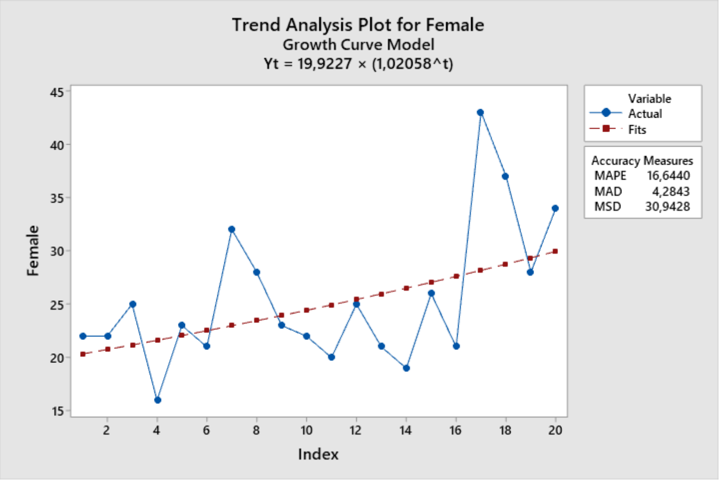

Model	Automatic Fitted-Curve	Accuracy Measurements		
		MAPE	MAD	MSD
Linear model	$Yt = 19.38 + 0.573t$	17.9781	4.5135	31.0256
**Quadratic model***	$\mathbf{Yt = 24.16 - 0.729t + 0.0620t^2}$	**15.4506**	**3.9259**	**27.6543**
Growth curve model	$Yt = 19.9227 \times (1.02058^t)$	16.6440	4.2843	30.9428

**The best-fitted model is the one that has the lowest value for three parameters (MAPE, MAD, and MSD), or at least for two parameters out of three, or at least having the lowest value for MAPE.*

2). Residual analysis, goodness-of-fit, and homoscedasticity for the Quadratic model

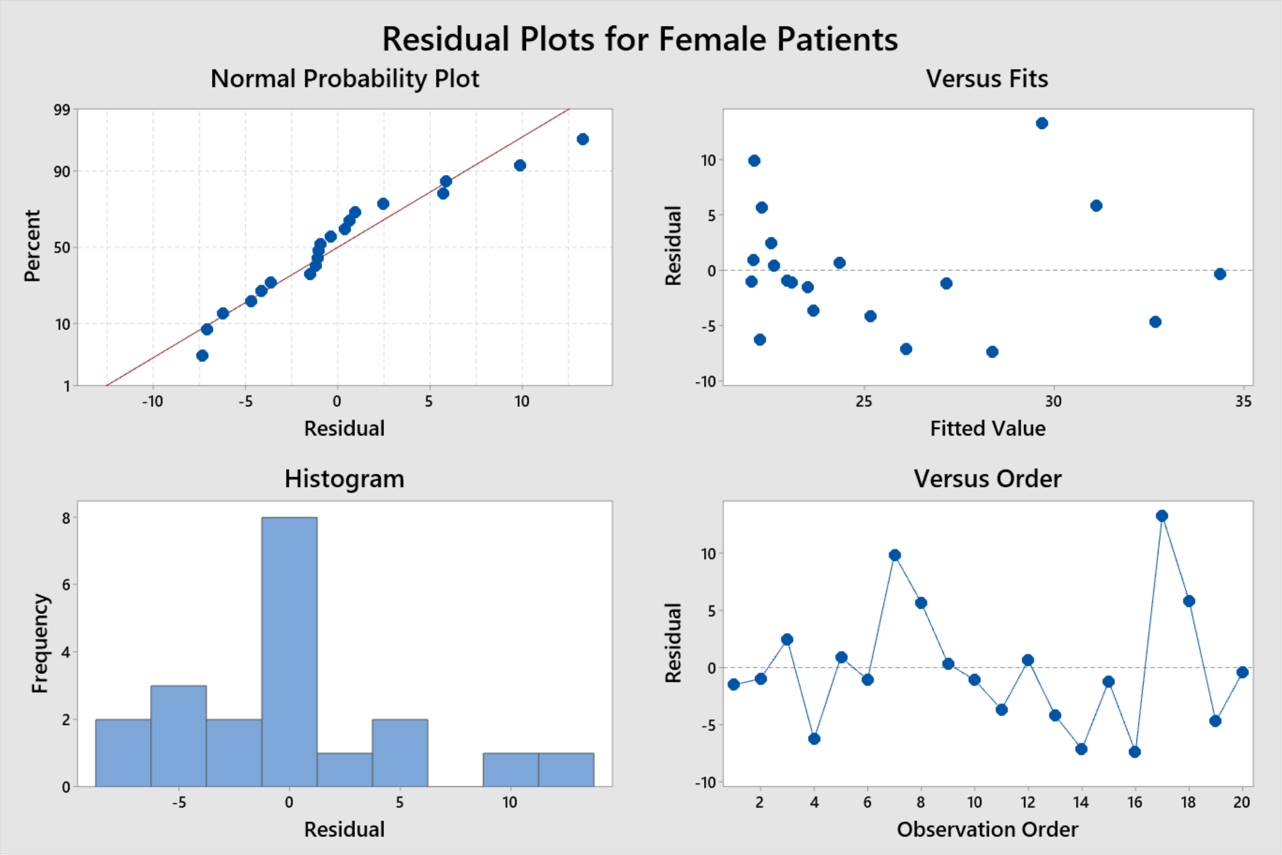

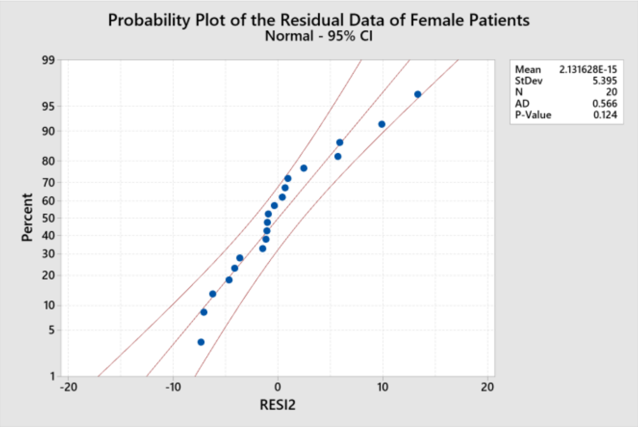

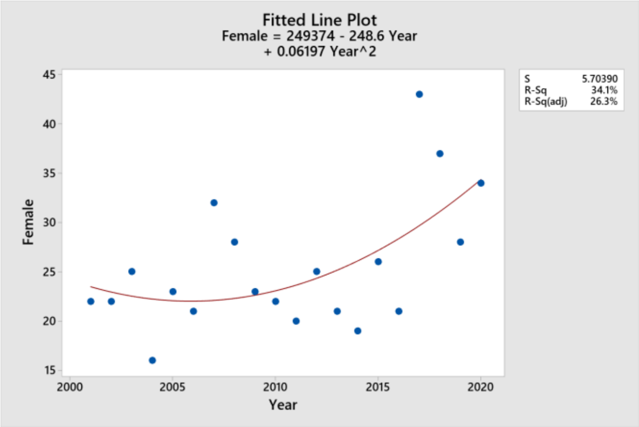

Interpretation:

- The normality test for the residuals yielded a p-value of **0.124,** suggesting a close alignment with the red line ("zero line" or "residuals mean line" or "identity line")and indicating a normal distribution.
- The R-square value for the Quadratic model is **34.1%**, indicating a reasonable fit of the regression model to the data.
- The plot of fitted values versus residuals shows consistent variance across all fitted values, suggesting the assumption of homoscedasticity is likely satisfied.

3). Autocorrelataion test

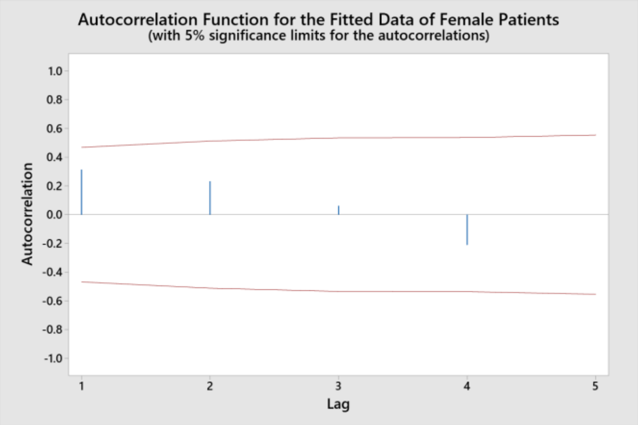

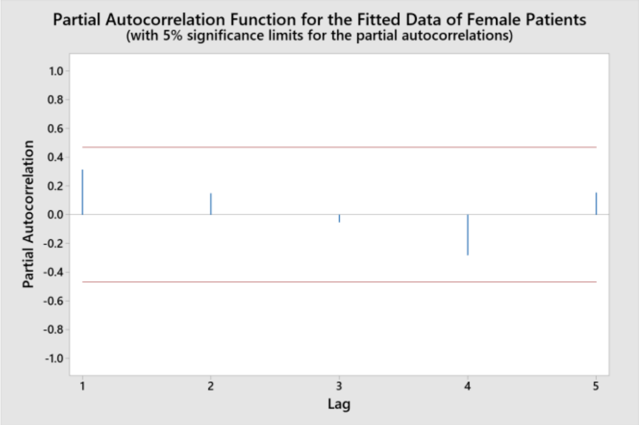

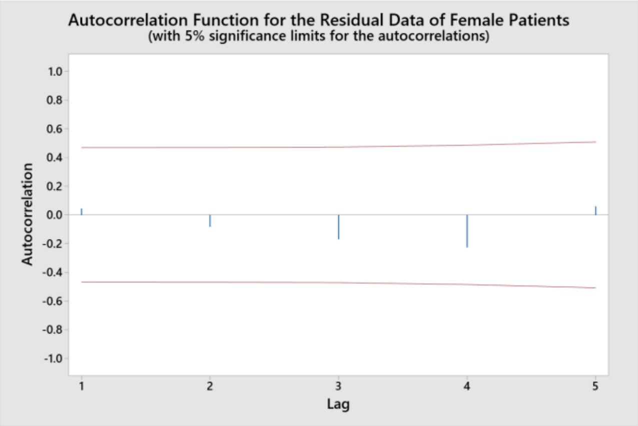

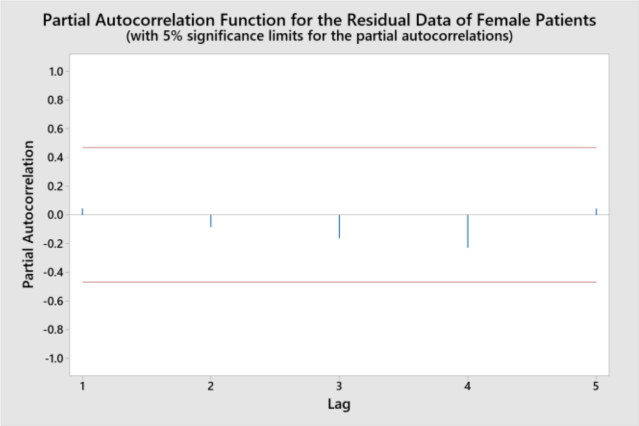

Interpretation:

The fitted and residual data of **female patients** show a correlation **near zero**, indicating stationarity, independence, and absence of systematic patterns. This suggests that the model effectively captures underlying data patterns and produces accurate forecasts.

4). The results of the significance test for the slope of the Quadratic curve estimation

ANOVA

	Sum of Squares	df	Mean Square	F	Sig.
Regression	218.597	1	218.597	6.344	**0.021**
Residual	620.203	18	34.456		
Total	838.800	19			

The independent variable is the Year.

5). Forecasting the number of cases for the next five years using the Quadratic model

Year	Forecasted Cases
2021	36.1877
2022	38.1241
2023	40.1843
2024	42.3686
2025	44.6768
**Mean**	40.3083
**Total**	241.8498
